# Thoracic and cardiovascular surgeries in Japan during 2021: Annual report by the Japanese Association for Thoracic Surgery

**DOI:** 10.1007/s11748-023-01997-6

**Published:** 2024-02-29

**Authors:** Committee for Scientific Affairs, The Japanese Association for Thoracic Surgery, Naoki Yoshimura, Yukio Sato, Hiroya Takeuchi, Tomonobu Abe, Shunsuke Endo, Yasutaka Hirata, Michiko Ishida, Hisashi Iwata, Takashi Kamei, Nobuyoshi Kawaharada, Shunsuke Kawamoto, Kohji Kohno, Hiraku Kumamaru, Kenji Minatoya, Noboru Motomura, Rie Nakahara, Morihito Okada, Hisashi Saji, Aya Saito, Masanori Tsuchida, Kenji Suzuki, Hirofumi Takemura, Tsuyoshi Taketani, Yasushi Toh, Wataru Tatsuishi, Hiroyuki Yamamoto, Takushi Yasuda, Masayuki Watanabe, Goro Matsumiya, Yoshiki Sawa, Hideyuki Shimizu, Masayuki Chida

**Affiliations:** 1 Committee for Scientific Affairs, The Japanese Association for Thoracic Surgery, Tokyo, Japan; 2 Department of Thoracic and Cardiovascular Surgery, Graduate School of Medicine, University of Toyama, Toyama, Japan; 3 Department of Thoracic Surgery, University of Tsukuba, Tsukuba, Japan; 4 Department of Surgery, Hamamatsu University School of Medicine, Shizuoka, Japan; 5 Division of Cardiovascular Surgery, Department of General Surgical Science, Gunma University, Maebashi, Japan; 6 Thoracic Surgery, Jichi Medical University Saitama Medical Center, Omiya, Japan; 7 Department of Cardiac Surgery, The University of Tokyo Hospital, Tokyo, Japan; 8 Cardiac Surgery, Handa City Hospita, Aichi, Japan; 9 Department of General Thoracic Surgery, Gifu University Hospital, Gifu, Japan; 10 Department of Surgery, Graduate School of Medicine, Tohoku University, Sendai, Japan; 11 Department of Cardiovascular Surgery, Sapporo Medical University School of Medicine, Sapporo, Japan; 12 Department of Cardiovascular Surgery, Tohoku Medical and Pharmaceutical University Hospital, Sendai, Japan; 13 Department of Gastrointestinal Tract Surgery, Fukushima Medical University, Fukushima, Japan; 14 Department of Healthcare Quality Assessment, Graduate School of Medicine, The University of Tokyo, Tokyo, Japan; 15 Department of Cardiovascular Surgery, Graduate School of Medicine, Kyoto University, Kyoto, Japan; 16 Department of Cardiovascular Surgery, Toho University Sakura Medical Center, Chiba, Japan; 17 Division of Thoracic Surgery, Tochigi Cancer Center, Tochigi, Japan; 18 Surgical Oncology, Hiroshima University, Hiroshima, Japan; 19 Department of Chest Surgery, St. Marianna University School of Medicine, Kawasaki, Japan; 20 Department of Surgery, Graduate School of Medicine, Yokohama City University, Yokohama, Japan; 21 Division of Thoracic and Cardiovascular Surgery, Niigata University Graduate School of Medical and Dental Sciences, Niigata, Japan; 22 Department of General Thoracic Surgery, Juntendo University School of Medicine, Tokyo, Japan; 23 Department of Cardiovascular Surgery, Kanazawa University, Kanazawa, Japan; 24 Department of Cardiovascular Surgery, Mitsui Memorial Hospital, Tokyo, Japan; 25 Department of Gastroenterological Surgery, National Hospital Organization Kyushu Cancer Center, Fukuoka, Japan; 26 Department of Surgery, Faculty of Medicine, Kindai University, Osaka, Japan; 27 Department of Gastroenterological Surgery, Cancer Institute Hospita, Tokyo, Japan; 28 Department of Cardiovascular Surgery, Chiba University Graduate School of Medicine, Chiba, Japan; 29 Graduate School of Medicine, Osaka Police Hospital, Osaka University, Osaka, Japan; 30 Department of Cardiovascular Surgery, Keio University, Tokyo, Japan; 31 Department of General Thoracic Surgery, Dokkyo Medical University, Tochigi, Japan

Since 1986, the Japanese Association for Thoracic Surgery (JATS) has conducted annual thoracic surgery surveys throughout Japan to determine statistics on the number of procedures performed by surgical categories. Herein, we summarize the results of the association’s annual thoracic surgery surveys in 2021.

Adhering to the norm thus far, thoracic surgery had been classified into three categories, including cardiovascular, general thoracic, and esophageal surgeries, with patient data for each group being examined and analyzed. We honor and value all members’ continued professional support and contributions.

Incidence of hospital mortality was included in the survey to determine nationwide status, which has contributed to Japanese surgeons’ understanding of the present status of thoracic surgery in Japan while helping in surgical outcome improvements by enabling comparisons between their work and that of others. This approach has enabled the association to gain a better understanding of present problems and prospects, which is reflected in its activities and member education.

The 30-day mortality (also known as *operative mortality*) is defined as death within 30 days of surgery, regardless of the patient’s geographic location, including post-discharge from the hospital. *Hospital mortality* is defined as death within any time interval following surgery among patients yet to be discharged from the hospital.

Transfer to a nursing home or a rehabilitation unit is considered hospital discharge unless the patient subsequently dies of complications from surgery, while hospital-to-hospital transfer during esophageal surgery is not considered a form of discharge. In contrast, hospital-to-hospital transfer 30 days following cardiovascular and general thoracic surgeries are considered discharge given that National Clinical Database (NCD)-related data were used in these categories.

Severe Acute Respiratory Syndrpme Coronavirus-2 (SARS-CoV-2), the causative pathogen for the coronavirus disease 2019 (COVID-19), first emerged in Wuhan, China, in December 2019 and by March 2020, it was declared a pandemic [1]. The pandemic of SARS-CoV-2 resulted in a global healthcare and financial crisis. There was a significant estimated reduction in national case volume of cardiovascular, general thoracic, and esophageal surgeries in Japan during 2020 [2–4]. We have to continue the estimation of the nationwide effect of SARS-CoV-2 pandemic on thoracic surgery in Japan, with surgical volume, outcomes and patient data for each group.

## Survey abstract

All data on cardiovascular, general thoracic, and esophageal surgeries were obtained from the NCD. In 2018, the data collection method for general thoracic and esophageal surgeries had been modified from self-reports using questionnaire sheets following each institution belonging to the JATS to an automatic package downloaded from the NCD in Japan.

The data collection related to cardiovascular surgery (initially self-reported using questionnaire sheets in each participating institution up to 2014) changed to downloading an automatic package from the Japanese Cardiovascular Surgery Database (JCVSD), which is a cardiovascular subsection of the NCD in 2015.

## Final report: 2021

### (A) Cardiovascular surgery

We are extremely pleased with the cooperation of our colleagues (members) in completing the cardiovascular surgery survey, which has undoubtedly improved the quality of this annual report. We are truly grateful for the significant efforts made by all participants within each participating institution in completing the JCVSD/NCD.

**Fig. 1:**
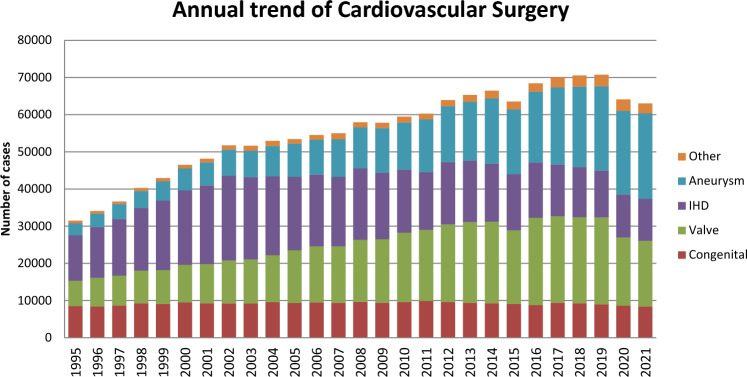
Annual trend of cardiovascular surgery

Figure 1 illustrates the development of cardiovascular surgery in Japan over the past 35 years. Aneurysm surgery includes only surgeries for thoracic and thoracoabdominal aortic aneurysms. Extra-anatomic bypass surgery for thoracic aneurysm and pacemaker implantation have been excluded from the survey since 2015. Assist device implantations were not included in the total number of surgical procedures but were included in the survey.

A total of 63,054 cardiovascular surgeries, including 59 heart transplants, had been performed in 2021, with a 1.6% decrease compared to that in 2020 (*n* = 64,075) [3]. Following on from 2020, a decline in the number of cases has been observed for the second consecutive year. Although the impact of the COVID-19 pandemic is suggested, verification from various perspectives is necessary.

Compared to data for 2020 [3] and 2011 [5], data for 2021 showed 2.9% (8349 vs. 8595) and 15.3% fewer surgeries for congenital heart disease, 3.8% (17,661 vs. 18,366) fewer and 7.8% fewer surgeries for valvular heart disease, 1.4% (11,364 vs. 11,524) and 27.1% fewer surgeries for ischemic heart procedures, and 2.0% (22,982 vs. 22,540) more and 62.7% more surgeries for thoracic aortic aneurysm, respectively. Data for individual categories are summarized in Tables 1, 2, 3, 4, 5, and 6.

**Table 1:** Congenital (total; 8349) (1) CPB ( +) (total; 6510)

	Neonate				Infant				1 ~ 17 years				≥ 18 years				Total			
	Cases	30-day mortality		Hospital mortality	Cases	30-day mortality		Hospital mortality	Cases	30-day mortality		Hospital mortality	Cases	30-day mortality		Hospital mortality	Cases	30-day mortality		Hospital mortality
		Hospital	After discharge			Hospital	After discharge			Hospital	*After discharge*			Hospital	After discharge			Hospital	After discharge	
PDA	6	0	0	0	5	1 (20.0)	0	1 (20.0)	3	0	0	0	14	0	0	0	28	1 (3.6)	0	1 (3.6)
Coarctation (simple)	9	0	0	1 (11.1)	14	0	0	0	9	0	0	0	12	0	0	0	44	0	0	1 (2.3)
+ VSD	39	0	0	0	46	0	0	0	10	0	0	0	1	0	0	0	96	0	0	0
+ DORV	3	0	0	0	4	0	0	0	4	0	0	0	0	0	0	0	11	0	0	0
+ AVSD	5	0	0	0	5	0	0	1 (20.0)	1	0	0	0	0	0	0	0	11	0	0	1 (9.1)
+ TGA	1	0	0	0	1	0	0	0	1	0	0	0	0	0	0	0	3	0	0	0
+ SV	2	0	0	0	9	1 (11.1)	0	1 (11.1)	3	0	0	0	0	0	0	0	14	1 (7.1)	0	1 (7.1)
+ Others	7	0	0	0	4	0	0	0	5	0	0	0	1	0	0	0	17	0	0	0
Interrupt. of Ao (simple)	0	0	0	0	1	0	0	0	0	0	0	0	0	0	0	0	1	0	0	0
+ VSD	26	1 (3.8)	0	2 (7.7)	24	1 (4.2)	0	2 (8.3)	14	0	0	0	0	0	0	0	64	2 (3.1)	0	4 (6.3)
+ DORV	0	0	0	0	2	0	0	0	0	0	0	0	0	0	0	0	2	0	0	0
+ Truncus	2	0	0	0	3	0	0	0	3	0	0	0	0	0	0	0	8	0	0	0
+ TGA	0	0	0	0	0	0	0	0	0	0	0	0	0	0	0	0	0	0	0	0
+ Others	2	0	0	0	2	0	0	0	3	0	0	0	0	0	0	0	7	0	0	0
Vascular ring	0	0	0	0	1	0	0	0	0	0	0	0	0	0	0	0	1	0	0	0
PS	6	0	0	0	30	0	0	0	62	0	0	0	17	0	0	0	115	0	0	0
PA・IVS or critical PS	5	0	0	0	37	0	0	0	57	0	0	0	9	0	0	0	108	0	0	0
TAPVR	104	4 (3.8)	0	9 (8.7)	43	1 (2.3)	0	2 (4.7)	11	0	0	0	0	0	0	0	158	5 (3.2)	0	11 (7.0)
PAPVR ± ASD	1	0	0	0	2	0	0	0	45	0	0	0	12	0	0	0	60	0	0	0
ASD	0	0	0	0	48	1 (2.1)	0	1 (2.1)	466	0	0	1 (0.2)	788	7 (0.9)	0	7 (0.9)	1302	8 (0.6)	0	9 (0.7)
Cor triatriatum	3	0	0	0	13	0	0	0	6	0	0	0	2	0	0	0	24	0	0	0
AVSD (partial)	0	0	0	0	7	0	0	0	35	0	0	0	13	0	0	0	55	0	0	0
AVSD (complete)	1	0	0	0	87	1 (1.1)	0	2 (2.3)	104	1 (1.0)	0	2 (1.9)	4	0	0	0	196	2 (1.0)	0	4 (2.0)
+ TOF or DORV	0	0	0	0	5	0	0	0	10	0	0	0	2	0	0	0	17	0	0	0
+ Others	0	0	0	0	0	0	0	0	0	0	0	0	0	0	0	0	0	0	0	0
VSD (subarterial)	1	0	0	0	77	0	0	0	138	0	0	0	8	0	0	0	224	0	0	0
VSD (perimemb./muscular)	6	0	0	0	645	0	0	0	331	0	0	1 (0.3)	17	0	0	0	999	0	0	1 (0.1)
VSD (type unknown)	0	0	0	0	0	0	0	0	3	0	0	0	112	2 (1.8)	0	2 (1.8)	115	2 (1.7)	0	2 (1.7)
VSD + PS	1	0	0	0	31	0	0	0	10	0	0	0	0	0	0	0	42	0	0	0
DCRV ± VSD	0	0	0	0	9	0	0	0	10	0	0	0	12	0	0	0	31	0	0	0
Aneurysm of sinus of Valsalva	0	0	0	0	0	0	0	0	1	0	0	0	0	0	0	0	1	0	0	0
TOF	14	0	0	0	157	0	0	1 (0.6)	157	0	0	0	57	0	0	1 (1.8)	385	0	0	2 (0.5)
PA + VSD	7	0	0	1 (14.3)	85	0	0	0	107	2 (1.9)	0	3 (2.8)	10	0	0	0	209	2 (1.0)	0	4 (1.9)
DORV	13	0	0	2 (15.4)	117	2 (1.7)	0	7 (6.0)	136	1 (0.7)	0	1 (0.7)	4	0	0	0	270	3 (1.1)	0	10 (3.7)
TGA (simple)	83	1 (1.2)	0	4 (4.8)	8	0	0	1 (12.5)	3	0	0	0	6	0	0	0	100	1 (1.0)	0	5 (5.0)
+ VSD	34	1 (2.9)	0	1 (2.9)	10	0	0	0	13	0	0	0	1	0	0	0	58	1 (1.7)	0	1 (1.7)
VSD + PS	0	0	0	0	1	0	0	0	0	0	0	0	1	0	0	0	2	0	0	0
Corrected TGA	1	0	0	0	7	0	0	0	33	0	0	0	10	0	0	0	51	0	0	0
Truncus arteriosus	5	0	0	1 (20.0)	16	0	0	0	27	0	0	0	2	0	0	0	50	0	0	1 (2.0)
SV	16	2 (12.5)	0	2 (12.5)	141	2 (1.4)	0	6 (4.3)	188	0	0	4 (2.1)	19	0	0	1 (5.3)	364	4 (1.1)	0	13 (3.6)
TA	4	0	0	0	33	0	0	0	48	0	0	0	3	0	0	0	88	0	0	0
HLHS	30	3 (10.0)	0	7 (23.3)	101	5 (5.0)	0	9 (8.9)	80	0	0	1 (1.3)	1	0	0	0	212	8 (3.8)	0	17 (8.0)
Aortic valve lesion	5	0	0	1 (20.0)	17	2 (11.8)	0	2 (11.8)	110	1 (0.9)	0	1 (0.9)	43	0	0	0	175	3 (1.7)	0	4 (2.3)
Mitral valve lesion	0	0	0	0	28	0	0	1 (3.6)	76	0	0	0	21	0	0	2 (9.5)	125	0	0	3 (2.4)
Ebstein	12	2 (16.7)	0	2 (16.7)	10	1 (10.0)	0	1 (10.0)	21	0	0	0	15	0	0	0	58	3 (5.2)	0	3 (5.2)
Coronary disease	0	0	0	0	3	0	0	0	21	0	0	0	2	0	0	0	26	0	0	0
Others	10	1 (10.0)	0	1 (10.0)	27	0	0	3 (11.1)	53	1 (1.9)	0	1 (1.9)	237	1 (0.4)	0	1 (0.4)	327	3 (0.9)	0	6 (1.8)
Conduit failure	0	0	0	0	1	0	0	0	20	0	0	0	9	0	0	0	30	0	0	0
Redo (excluding conduit failure)	1	0	0	0	50	1 (2.0)	0	2 (4.0)	106	2 (1.9)	0	2 (1.9)	69	0	0	0	226	3 (1.3)	0	4 (1.8)
Total	465	15 (3.2)	0	34 (7.3)	1967	19 (1.0)	0	43 (2.2)	2544	8 (0.3)	0	17 (0.7)	1534	10 (0.7)	0	14 (0.9)	6510	52 (0.8)	0	108 (1.7)
(), % mortality																				
*CPB* cardiopulmonary bypass; *PDA* patent ductus arteriosus; *VSD* ventricular septal defect; *DORV* double outlet right ventricle; *AVSD* atrioventricular septal defect; *TGA* transposition of great arteries; *SV* single ventricle; *Interrupt. of Ao.* interruption of aorta; *PS* pulmonary stenosis; *PA-IVS* pulmonary atresia with intact ventricular septum; *TAPVR* total anomalous pulmonary venous return; *PAPVR* partial anomalous pulmonary venous return; *ASD* atrial septal defect; *TOF* tetralogy of Fallot; *DCRV* double-chambered right ventricle; *TA* tricuspid atresia; *HLHS* hypoplastic left heart syndrome; *RV-PA* right ventricle-pulmonary artery																				

(2) CPB (−) (total; 1839)																				
	Neonate				Infant				1–17 years				≥ 18 years				Total			
	Cases	30-day mortality		Hospital mortality	Cases	30-day mortality		Hospital mortality	Cases	30-day mortality		Hospital mortality	Cases	30-day mortality		Hospital mortality	Cases	30-day mortality		Hospital mortality
		Hospital	After discharge			Hospital	After discharge			Hospital	After discharge			Hospital	After discharge			Hospital	After discharge	
PDA	230	7 (3.0)	0	11 (4.8)	130	1 (0.8)	0	6 (4.6)	6	0	0	0	4	0	0	0	370	8 (2.2)	0	17 (4.6)
Coarctation (simple)	6	0	0	1 (16.7)	7	0	0	0	1	0	0	0	5	0	0	0	19	0	0	1 (5.3)
+ VSD	40	2 (5.0)	0	2 (5.0)	12	0	0	0	0	0	0	0	0	0	0	0	52	2 (3.8)	0	2 (3.8)
+ DORV	4	0	0	0	3	0	0	0	0	0	0	0	0	0	0	0	7	0	0	0
+ AVSD	8	0	0	0	1	0	0	0	1	0	0	0	0	0	0	0	10	0	0	0
+ TGA	1	0	0	0	0	0	0	0	0	0	0	0	0	0	0	0	1	0	0	0
+ SV	6	0	0	1 (16.7)	0	0	0	0	0	0	0	0	0	0	0	0	6	0	0	1 (16.7)
+ Others	3	0	0	1 (33.3)	6	0	0	0	0	0	0	0	0	0	0	0	9	0	0	1 (11.1)
Interrupt. of Ao (simple)	0	0	0	0	0	0	0	0	0	0	0	0	0	0	0	0	0	0	0	0
+ VSD	21	0	0	2 (9.5)	5	0	0	0	1	0	0	0	0	0	0	0	27	0	0	2 (7.4)
+ DORV	1	0	0	0	0	0	0	0	0	0	0	0	0	0	0	0	1	0	0	0
+ Truncus	6	0	0	0	1	0	0	0	0	0	0	0	0	0	0	0	7	0	0	0
+ TGA	0	0	0	0	0	0	0	0	0	0	0	0	0	0	0	0	0	0	0	0
+ Others	2	0	0	0	1	0	0	0	4	0	0	0	0	0	0	0	7	0	0	0
Vascular ring	0	0	0	0	20	0	0	0	6	0	0	0	0	0	0	0	26	0	0	0
PS	1	0	0	0	3	0	0	0	4	0	0	0	0	0	0	0	8	0	0	0
PA・IVS or Critical PS	14	0	0	1 (7.1)	13	1 (7.7)	0	1 (7.7)	1	0	0	0	1	0	0	0	29	1 (3.4)	0	2 (6.9)
TAPVR	8	1 (12.5)	0	2 (25.0)	5	0	0	0	1	0	0	0	0	0	0	0	14	1 (7.1)	0	2 (14.3)
PAPVR ± ASD	0	0	0	0	1	0	0	0	0	0	0	0	0	0	0	0	1	0	0	0
ASD	1	0	0	0	3	0	0	0	4	0	0	0	0	0	0	0	8	0	0	0
Cor triatriatum	0	0	0	0	0	0	0	0	0	0	0	0	0	0	0	0	0	0	0	0
AVSD (partial)	1	0	0	0	1	0	1 (100.0)	0	6	0	0	0	1	0	0	0	9	0	1(11.1)	0
AVSD (complete)	37	0	0	0	70	1 (1.4)	0	3 (4.3)	8	0	0	0	2	0	0	0	117	1 (0.9)	0	3 (2.6)
+ TOF or DORV	0	0	0	0	3	0	0	0	1	0	0	0	0	0	0	0	4	0	0	0
+ Others	0	0	0	0	0	0	0	0	0	0	0	0	0	0	0	0	0	0	0	0
VSD (subarterial)	4	0	0	0	9	0	0	0	0	0	0	0	0	0	0	0	13	0	0	0
VSD (perimemb./muscular)	58	1 (1.7)	0	4 (6.9)	143	3 (2.1)	0	4 (2.8)	4	0	0	0	2	0	0	0	207	4 (1.9)	0	8 (3.9)
VSD (Type Unknown)	0	0	0	0	0		0	0	0	0	0	0	0	0	0	0	0	0	0	0
VSD + PS	1	0	0	0	3	0	0	0	0	0	0	0	0	0	0	0	4	0	0	0
DCRV ± VSD	0	0	0	0	0	0	0	0	0	0	0	0	0	0	0	0	0	0	0	0
Aneurysm of sinus of Valsalva	0	0	0	0	0	0	0	0	0	0	0	0	0	0	0	0	0	0	0	0
TOF	9	0	0	0	50	0	0	0	4	0	0	0	0	0	0	0	63	0	0	0
PA + VSD	22	0	0	1 (4.5)	35	1 (2.9)	0	2 (5.7)	7	1 (14.3)	0	1 (14.3)	0	0	0	0	64	2 (3.1)	0	4 (6.3)
DORV	47	0	0	3 (6.4)	58	0	0	2 (3.4)	6	0	0	0	1	1(100.0)	0	1(100.0)	112	1 (0.9)	0	6 (5.4)
TGA (simple)	10	0	0	0	6	0	0	0	0	0	0	0	0	0	0	0	16	0	0	0
+ VSD	8	1 (12.5)	0	1 (12.5)	2	0	0	0	1	0	0	0	0	0	0	0	11	1 (9.1)	0	1 (9.1)
VSD + PS	0	0	0	0	0	0	0	0	0	0	0	0	0	0	0	0	0	0	0	0
Corrected TGA	5	0 0	0	0	7	0	0	0	10	0	0	0	6	0	0	0	28	0	0	0
Truncus arteriosus	11		0	1 (9.1)	1	0	0	0	0	0	0	0	0	0	0	0	12	0	0	1 (8.3)
SV	40	0	0	3 (7.5)	58	3 (5.2)	0	6 (10.3)	8	0	0	0	3	0	0	2(66.7)	109	3 (2.8)	0	11 (10.1)
TA	22	0	0	0	12	0	0	0	8	0	0	0	3	0	0	0	45	0	0	0
HLHS	66	1 (1.5)	0	8 (12.1)	27	1 (3.7)	1 (3.7)	1 (3.7)	11	0	0	1 (9.1)	1	0	0	0	105	2 (1.9)	1(1.0)	10 (9.5)
Aortic valve lesion	7	0	0	0	9	1 (11.1)	0	1 (11.1)	4	0	0	0	0	0	0	0	20	1 (5.0)	0	1 (5.0)
Mitral valve lesion	3	0	0	0	1	0	0	0	1	0	0	0	0	0	0	0	5	0	0	0
Ebstein	10	1 (10.0)	0	1 (10.0)	3	1 (33.3)	0	1 (33.3)	0	0	0	0	0	0	0	0	13	2 (15.4)	0	2 (15.4)
Coronary disease	0	0	0	0	1	0	0	0	0	0	0	0	0	0	0	0	1	0	0	0
Others	12	1 (8.3)	0	2 (16.7)	8	0	0	1 (12.5)	16	3 (18.8)	0	5 (31.3)	1	0	0	0	37	4 (10.8)	0	8 (21.6)
Conduit failure	0	0	0	0	0	0	0	0	0	0	0	0	0	0	0	0	0	0	0	0
Redo (excluding conduit failure)	14	0	0	0	69	2 (2.9)	0	3 (4.3)	120	5 (4.2)	0	8 (6.7)	39	1(2.6)	0	2(5.1)	242	8 (3.3)	0	13 (5.4)
Total	739	15 (2.0)	0	45 (6.1)	787	15 (1.9)	2 (0.3)	31 (3.9)	244	9 (3.7)	0	15 (6.1)	69	2(2.9)	0	5(7.2)	1839	41 (2.2)	2(0.11)	96 (5.2)
(), % mortality																				
*CPB* cardiopulmonary bypass; *PDA* patent ductus arteriosus; *VSD* ventricular septal defect; *DORV* double outlet right ventricle; *AVSD* atrioventricular septal defect; *TGA* transposition of the great arteries; *SV* single ventricle; *Interrupt. of Ao.* interruption of aorta; *PS* pulmonary stenosis; *PA-IVS* pulmonary atresia with intact ventricular septum; *TAPVR* total anomalous pulmonary venous return; *PAPVR* partial anomalous pulmonary venous return; *ASD* atrial septal defect; *TOF* tetralogy of Fallot; *DCRV* double-chambered right ventricle; *TA* tricuspid atresia; *HLHS* hypoplastic left heart syndrome; *RV-PA* right ventricle-pulmonary artery																				

(3) Main procedure																					
		Neonate				Infant				1- 17 years				≥ 18 years				Total			
		Cases	30-day mortality			Cases	30-day mortality			Cases	30-day mortality			Cases	30-day mortality			Cases	30-day mortality		
				After discharge	Hospital mortality		Hospital	After discharge	Hospital mortality		Hospital	After discharge	Hospital mortality		Hospital	After discharge	Hospital mortality		Hospital	After discharge	Hospital mortality
1	SP Shunt	100	0	0	3 (3.0)	267	0	0	6 (2.2)	37	0	0	0	1	0	0	0	405	0	0	9 (2.2)
2	PAB	236	1 (0.4)	0	9 (3.8)	314	4 (1.3)	1 (0.3)	8 (2.5)	9	0	0	0	0	0	0	0	559	5 (0.9)	1 (0.2)	17 (3.0)
3	Bidirectional Glenn or hemi-Fontan ± α	0	0	0	0	217	2 (0.9)	0	6 (2.8)	73	0	0	1 (1.4)	1	0	0	0	291	2 (0.7)	0	7 (2.4)
4	Damus-Kaye-Stansel operation	0	0	0	0	17	0	0	1 (5.9)	6	0	0	0	0	0	0	0	23	0	0	1 (4.3)
5	PA reconstruction/repair (including redo)	13	1 (7.7)	0	1 (7.7)	179	2 (1.1)	0	4 (2.2)	194	2 (1.0)	0	4 (2.1)	23	1 (4.3)	0	1 (4.3)	409	6 (1.5)	0	10 (2.4)
6	RVOT reconstruction/repair	6	1 (16.7)	0	1 (16.7)	224	1 (0.4)	0	3 (1.3)	267	2 (0.7)	0	3 (1.1)	42	0	0	0	539	4 (0.7)	0	7 (1.3)
7	Rastelli procedure	2	0	0	0	43	0	0	0	97	0	0	0	2	0	0	0	144	0	0	0
8	Arterial switch procedure	122	3 (2.5)	1 (0.8)	7 (5.7)	17	0	0	0	2	0	0	0	0	0	0	0	141	3 (2.1)	1 (0.7)	7 (5.0)
9	Atrial switch procedure	0	0	0	0	4	1 (25.0)	0	1 (25.0)	4	0	0	0	2	0	0	0	10	1 (10.0)	0	1 (10.0)
10	Double switch procedure	0	0	0	0	0	0	0	0	8	0	0	0	0	0	0	0	8	0	0	0
11	Repair of anomalous origin of CA	0	0	0	0	1	0	0	0	4	0	0	0	0	0	0	0	5	0	0	0
12	Closure of coronary AV fistula	0	0	0	0	0	0	0	0	1	0	0	0	0	0	0	0	1	0	0	0
13	Fontan / TCPC	0	0	0	0	1	0	0	0	356	0	0	2 (0.6)	28	0	0	1 (3.6)	385	0	0	3 (0.8)
14	Norwood procedure	28	2 (7.1)	0	5 (17.9)	76	4 (5.3)	0	7 (9.2)	0	0	0	0	0	0	0	0	104	6 (5.8)	0	12 (11.5)
15	Ventricular septation	0	0	0	0	0	0	0	0	0	0	0	0	0	0	0	0	0	0	0	0
16	Left side AV valve repair (including Redo)	0	0	0	0	43	0	0	1 (2.3)	84	0	0	0	28	0	0	1 (3.6)	155	0	0	2 (1.3)
17	Left side AV valve replace (including Redo)	0	0	0	0	11	0	0	0	36	0	0	0	20	0	0	1 (5.0)	67	0	0	1 (1.5)
18	Right side AV valve repair (including Redo)	18	2 (11.1)	0	2 (11.1)	71	1 (1.4)	0	4 (5.6)	85	0	0	0	64	0	0	0	238	3 (1.3)	0	6 (2.5)
19	Right side AV valve replace (including Redo)	0	0	0	0	1	0	0	0	8	0	0	0	24	0	0	0	33	0	0	0
20	Common AV valve repair (including Redo)	1	0	0	0	11	0	0	2 (18.2)	8	0	0	0	1	0	0	0	21	0	0	2 (9.5)
21	Common AV valve replace (including Redo)	0	0	0	0	2	1 (50.0)	0	1 (50.0)	14	1 (7.1)	0	2 (14.3)	8	0	0	0	24	2 (8.3)	0	3 (12.5)
22	Repair of supra-aortic stenosis	0	0	0	0	9	0	0	1 (11.1)	23	0	0	1 (4.3)	0	0	0	0	32	0	0	2 (6.3)
23	Repair of subaortic stenosis (including Redo)	0	0	0	0	7	0	0	0	41	0	0	0	3	0	0	0	51	0	0	0
24	Aortic valve plasty ± VSD Closure	2	0	0	0	5	0	0	0	36	0	0	0	6	0	0	0	49	0	0	0
25	Aortic valve replacement	0	0	0	0	2	0	0	0	28	0	0	0	36	0	0	0	66	0	0	0
26	AVR with annular enlargement	0	0	0	0	1	0	0	0	14	0	0	0	6	0	0	0	21	0	0	0
27	Aortic root Replace (except Ross)	0	0	0	0	1	1 (100.0)	0	1 (100.0)	8	1 (12.5)	0	1 (12.5)	17	0	0	0	26	2 (7.7)	0	2 (7.7)
28	Ross procedure	0	0	0	0	4	0	0	0	19	0	0	0	23	0	0	0				
29	Bilateral pulmonary artery banding	180	9 (5.0)	0	25 (13.9)	23	1 (4.3)	1 (4.3)	2 (8.7)	0	0	0	0	0	0	0	0	203	10 (4.9)	1 (0.5)	27 (13.3)
Total		708	19 (2.7)	1 (0.1)	53 (7.5)	1551	18 (1.2)	2 (0.1)	48 (3.1)	1462	6 (0.4)	0	14 (1.0)	312	1 (0.3)	0	4 (1.3)	4033	44 (1.1)	3 (0.07)	119 (3.0)
(), % mortality																					
*SP* systemic-pulmonary; *PAB* pulmonary artery banding; *PA* pulmonary artery; *RVOT* right ventricular outflow tract; *CA* coronary artery; *AV fistula* arteriovenous fistula; *TCPC* total cavopulmonary connection; *AV valve* atrioventricular valve; *VSD* ventricular septal defect; *AVR* aortic valve replacement

**Table 2:** Acquired (total, (1) + (2) + (4) + (5) + (6) + (7) + isolated operations for arrhythmia in (3); 31,479

(1) Valvelar heart disease (total; 17,661)																	
	Valve	Cases	Operation					30-Day mortality				Hospital mortality		Redo			
			Mechanical	Bioprosthesis	Repair	Unknown	With CABG	Hospital		After discharge				Cases	30-Day mortality		Hospital mortality
								Replace	Repair	Replace	Repair	Replace	Repair		Hospital	After discharge	
Isolated	A	8206	879	7142	113	72	2020	118 (1.5)	0	4 (0.05)	1 (0.9)	202 (2.5)	2 (1.8)	625	23 (3.7)	0	33 (5.3)
	M	4415	384	847	3155	29	557	63 (5.1)	21 (0.7)	0	2 (0.06)	97 (7.9)	39 (1.2)	527	18 (3.3)	1 (0.2)	34 (6.5)
	T	221	7	44	167	3	37	0	7 (4.2)	0	0	3 (5.9)	11 (6.6)	57	1 (1.8)	0	3 (5.3)
	P	20	0	17	0	3	1	0	0	0	0	0	0	16	0	0	0
A + M		971					152	42 (4.3)		1 (0.1)		74 (7.6)		143	11 (7.7)	0	17 (11.9)
	A		171	777	22	1											
	M		134	308	520	9											
A + T		366					50	6 (1.6)		0		16 (4.4)		55	2 (3.6)	0	4 (7.3)
	A		38	323	5	0											
	T		0	0	358	8											
M + T		2663					274	44 (1.7)		1 (0.04)		81 (3.0)		317	7 (2.2)	0	13 (4.1)
	M		255	750	1631	27											
	T		6	17	2622	18											
A + M + T		738					89	29 (3.9)		0		49 (6.6)		99	5 (5.1)	0	6 (6.1)
	A		100	624	12	2											
	M		96	278	356	8											
	T		3	6	728	1											
others		61					3	1 (1.6)		0		3 (4.9)		23	1 (4.3)	0	1 (4.3)
Total		17,661					3183	303 (1.7)		6 (0.03)		525 (3.0)		1862	68 (3.7)	1 (0.05)	111 (6.0)

	Cases	30-day mortality
TAVR	12,202	140 (1.1)

(2) Ischemic heart disease (total, (A) + (B); 11,364)																					
(A) Isolated CABG (total; (a) + (b); 10,184)																					
(a-1) On-pump arrest CABG (total; 2374)																					
	Primary, elective				Primary, emergent				Redo, elective				redo, emergent				Artery only	Artery + SVG	SVG only	others	Unclear
	Cases	30 day mortality		Hospital mortality	Cases	30 day mortality		Hospital mortality	Cases	30 day mortality		Hospital mortality	Cases	30 day mortality		Hospital mortality					
		Hospital	After discharge			Hospital	After discharge			Hospital	After discharge			Hospital	After discharge						
1VD	46	0 (0.0)	0	0 (0.0)	11	3 (27.3)	0	3 (27.3)	0	0	0	0	0	0	0	0	16	24	15	2	0
2VD	296	1 (0.3)	0	4 (1.4)	27	3 (11.1)	0	3 (11.1)	0	0	0	0	1	0 (0.0)	0	0 (0.0)	38	263	22	1	0
3VD	877	12 (1.4)	0	17 (1.9)	111	8 (7.2)	0	10 (9.0)	3	0 (0.0)	0	0 (0.0)	0	0	0	0	45	916	22	8	0
LMT	796	8 (1.0)	0	11 (1.4)	168	6 (3.6)	0	13 (7.7)	6	0	0	0	0	0	0	0	69	851	43	6	1
No info	21	1	0	1 (4.8)	8	1 (12.5)	0	4 (50.0)	1	0 (0.0)	0	1 (100.0)	2	1 (50.0)	0	1 (50.0)	3	15	11	1	2
Total	2036	22 (1.1)	0	33 (1.6)	325	21 (6.5)	0	33 (10.2)	10	0 (0.0)	0	1 (10.0)	3	1 (33.3)	0	1 (33.3)	171	2069	113	18	3
Kawasaki	4	1 (25.0)	0	0 (0.0)	0	0	0	0	0	0	0	0	0	0	0	0	4	0	0	0	0
on dialysis	250	10 (4.0)	0	14 (5.6)	36	4	0	6 (16.7)	3	0	0	1	0	0	0	0	20	252	16	1	0
(), % mortality																					
*CABG* coronary artery bypass grafting; *1VD* one-vessel disease; *2VD* two-vessel disease; *3VD* three-vessel disease; *LMT* left main trunk; *SVG* saphenous vein graft																					
LMT includes LMT alone or LMT with other branch diseases																					

(a-2) On-pump beating CABG (total; 2003)																					
	Primary, elective				Primary, emergent				Redo, elective				Redo, emergent				Artery only	Artery + SVG	SVG only	Others	Unclear
	Cases	30 day mortality		Hospital mortality	Cases	30 day mortality		Hospital mortality	Cases	30 day mortality		Hospital mortality	Cases	30 day mortality		Hospital mortality					
		Hospital	After discharge			Hospital	After discharge			Hospital	After discharge			Hospital	After discharge						
1VD	38	0 (0.0)	0 (0.0)	0 (0.0)	11	1 (9.1)	0	2 (18.2)	0	0	0	0	1	0	0	0	23	17	10	0	0
2VD	206	1 (0.5)	2 (1.0)	3 (1.5)	38	1 (2.6)	0	3 (7.9)	5	0	0	0	1	0	0	0	52	174	22	2	0
3VD	662	11 (1.7)	0 (0.0)	18 (2.7)	110	13 (11.8)	0	19 (17.3)	4	0	0	0 (0.0)	3	1 (33.3)	0	1 (33.3)	81	668	28	2	0
LMT	643	17 (2.6)	1 (0.2)	27 (4.2)	228	19 (8.3)	0	26 (11.4)	11	1 (9.1)	0	2 (18.2)	3	0	0	1 (33.3)	116	731	32	5	1
no info	27	0 (0.0)	0 (0.0)	1 (3.7)	9	1 (11.1)	0	1 (11.1)	0	0	0	0	3	0	0	1 (33.3)	14	18	7	0	0
Total	1576	29 (1.8)	3 (0.2)	49 (3.1)	396	35 (8.8)	0 (0.0)	51 (12.9)	20	1 (5.0)	0	2 (10.0)	11	1 (9.1)	0	3 (27.3)	286	1608	99	9	1
Kawasaki	2	0	0	0	2	0	0	0	2	0	0	0	0	0	0	0	3	2	1	0	0
on dialysis	255	11 (4.3)	1	21 (8.2)	59	9 (15.3)	0 (0.0)	11 (18.6)	6	1 (16.7)	0	2 (33.3)	1	1 (100.0)	0	1 (100.0)	23	278	19	1	0
(), % mortality																					
CABG, coronary artery bypass grafting; 1VD, one-vessel disease; 2VD two-vessel disease; 3VD, three-vessel disease; LMT, left main trunk; SVG, saphenous vein graft																					
LMT includes LMT alone or LMT with other branch diseases																					

(b) Off-pump CABG (total; 5807)																					
(Including cases of planned off-pump CABG in which, during surgery, the change is made to an on-pump CABG or on-pump beating-heart procedure)																					
	Primary, elective				Primary, emergent				Redo, elective				Redo, emergent				Artery only	Artery + SVG	SVG only	Others	Unclear
	Cases	30 day mortality		Hospital mortality	Cases	30 day mortality		Hospital mortality	Cases	30 day mortality		Hospital mortality	Cases	30 day mortality		Hospital mortality					
		Hospital	After discharge			Hospital	After discharge			Hospital	After discharge			Hospital	After discharge						
1VD	307	0 (0.0)	0	5 (1.6)	36	3 (8.3)	0	6 (16.7)	7	0	0	0	0	0	0	0	244	72	32	0	2
2VD	786	6 (0.8)	0	9 (1.1)	63	1 (1.6)	0	4 (6.3)	8	0	0	1 (12.5)	0	0	0	0	299	533	20	4	1
3VD	2093	21 (1.0)	1 (0.0)	35 (1.7)	186	5 (2.7)	0	11 (5.9)	14	0	0	0	1	0	0	0	462	1780	36	15	1
LMT	1873	14 (0.7)	1 (0.1)	26 (1.4)	331	10 (3.0)	1 (0.3)	18 (5.4)	13	0	0	1 (7.7)	2	0	0	0	603	1562	44	10	0
no info	67	1 (1.5)	0 (0.0)	1 (1.5)	15	1	0	1 (6.7)	4	0	0	0	1	1 (100.0)	0	1 (100.0)	34	45	7	1	0
Total	5126	42 (0.8)	2 (0.0)	76 (1.5)	631	20 (3.2)	1 (0.2)	40 (6.3)	46	0	0	2 (4.3)	4	1 (25.0)	0	1 (25.0)	1642	3992	139	30	4
Kawasaki	15	0	0	0	1	0	0	0	1	0	0	0	0	0	0	0	7	9	0	0	1
On dialysis	566	12 (2.1)	1 (0.2)	26 (4.6)	55	6 (10.9)	0	10 (18.2)	13	0	0	2 (15.4)	1	0	0	0	154	458	18	5	0
(), % mortality																					
*CABG* coronary artery bypass grafting; *1VD* one-vessel disease; *2VD* two-vessel disease; *3VD* three-vessel disease; *LMT* left main trunk; *SVG* saphenous vein graft																					
LMT includes LMT alone or LMT with other branch diseases																					

(c) Cases of conversion, during surgery, from off-pump CABG to on-pump CABG or on- pump beating-heart CABG (these cases are also included in category (b))																
	Primary, elective				Primary, emergent				Redo, elective				Redo, emergent			
	Cases	30 day mortality		Hospital mortality	Cases	30 day mortality		Hospital mortality	Cases	30 day mortality		Hospital mortality	Cases	30 day mortality		Hospital mortality
		Hospital	After discharge			Hospital	After discharge			Hospital	After discharge			Hospital	After discharge	
Converted to arrest	24	2 (8.3)	0	2 (8.3)	8	0 (0.0)	0	1 (12.5)	0	0	0	0	0	0	0	0
Converted to beating	107	5 (4.7)	0	6 (5.6)	17	3 (17.6)	0	6 (35.3)	1	0	0	0	0	0	0	0
Total	131	7 (5.3)	0	8 (6.1)	25	3 (12.0)	0	7 (28.0)	1	0	0	0	0	0	0	0
On dialysis	28	5 (17.9)	0	5 (17.9)	3	1 (33.3)	0	1 (33.3)	0	0	0	0	0	0	0	0
(), % mortality																
*CABG* coronary artery bypass grafting																

(B) Operation for complications of MI (total; 1180)											
	Chronic				Acute				Concomitant operation		
	Cases	30-day mortality		Hospital mortality	Cases	30-day mortality		Hospital mortality			
		Hospital	After discharge			Hospital	After discharge		CABG	MVP	MVR
Infarctectomy or Aneurysmectomy	90	6 (6.7)	0	8 (8.9)	36	10 (27.8)	1 (2.8)	15 (41.7)	56	14	6
VSP closure	86	12 (14.0)	0	21 (24.4)	244	60 (24.6)	0	77 (31.6)	85	9	0
Cardiac rupture	50	7 (14.0)	0	10 (20.0)	231	64 (27.7)	0	78 (33.8)	38	3	4
Mitral regurgitation											
(1) Papillary muscle rupture	23	2 (8.7)	0	2 (8.7)	70	14 (20.0)	0	20 (28.6)	35	15	78
(2) Ischemic	142	9 (6.3)	0	11 (7.7)	39	6 (15.4)	0	9 (23.1)	134	111	70
Others	90	1 (1.1)	0	2 (2.2)	79	20 (25.3)	0	25 (31.6)	59	10	4
Total	481	37 (7.7)	0	54 (11.2)	699	174 (24.9)	1 (0.1)	224 (32.0)	407	162	162
(), % mortality											
*MI* myocardial infarction; *CABG* coronary artery bypass grafting; *MVP* mitral valve repair; *MVR* mitral valve replacement; *VSP* ventricular septal perforation											
Acute, within 2 weeks from the onset of myocardial infarction											

(3) Operation for arrhythmia (total; 6720)											
	Cases	30-day mortality		Hospital mortality	Concomitant operation						
					Isolated	Congenital	Valve	IHD	Others	Multiple combination	
		Hospital	After discharge							2 categories	3 categories
Maze	3442	53 (1.5)	0	98 (2.8)	155	172	2918	572	319	660	36
For WPW	1	0	0	0	0	0	1	1	0	1	0
For ventricular tachyarrhythmia	23	0	0	1 (4.3)	1	1	4	10	5	3	0
Others	3254	74 (2.3)	2 (0.06)	127 (3.9)	80	153	2680	635	382	654	38
Total	6720	127 (1.9)	2 (0.03)	226 (3.4)	236	326	5603	1218	706	1318	74
(), % mortality											
*WPW* Wolff–Parkinson-White syndrome; *IHD* ischemic heart disease											
Except for 170 isolated cases, all remaining 5164 cases are doubly allocated, one for this subgroup and the other for the subgroup corresponding to the concomitant operations											

(4) Operation for constrictive pericarditis (total; 190)								
	CPB ( +)				CPB ( −)			
	Cases	30-day mortality		Hospital mortality	Cases	30-day mortality		Hospital mortality
		Hospital	After discharge			Hospital	After discharge	
Total	102	6 (5.9)	0	12 (11.8)	88	4 (4.5)	2 (2.3)	7 (8.0)
(), % mortality								
*CPB* cardiopulmonary bypass								

(5) Cardiac tumor (total; 618)								
	Cases	30-day mortality		Hospital mortality	Concomitant operation			
		Hospital	After discharge		AVR	MVR	CABG	Others
Benign tumor	550	6 (1.1)	0	8 (1.5)	24	15	40	120
(Cardiac myxoma)	392	2 (0.5)	0	3 (0.8)	7	3	22	73
Malignant tumor	68	8 (11.8)	0	8 (11.8)	0	2	7	13
(Primary)	38	3 (7.9)	0	3 (7.9)	0	1	4	8
(), % mortality								
*AVR* aortic valve replacement; *MVR* mitral valve replacement; *CABG* coronary artery bypass grafting								

(6) HOCM and DCM (total; 226)								
	Cases	30-day mortality		Hospital mortality	Concomitant operation			
		Hospital	After discharge		AVR	MVR	MVP	CABG
Myectomy	116	4 (3.4)	0	5 (4.3)	41	16	14	6
Myotomy	4	0	0	0	1	1	0	0
No-resection	100	7 (7.0)	0	13 (13.0)	20	51	49	4
Volume reduction surgery of the left ventricle	6	0	0	1 (16.7)	1	0	4	1
Total	226	11 (4.9)	0	19 (8.4)	63	68	67	11
(), % mortality								
*HOCM* hypertrophic obstructive cardiomyopathy; *DCM* dilated cardiomyopathy; *AVR* aortic valve replacement; *MVR* mitral valve replacement; *MVP* mitral valve repair, *CABG* coronary artery bypass grafting								

(7) Other open-heart operation (total; 1184)				
	Cases	30-day mortality		Hospital mortality
		Hospital	After discharge	
Open-heart operation	491	57 (11.6)	0	82 (16.7)
Non-open-heart operation	693	81 (11.7)	0	115 (16.6)
Total	1184	138 (11.7)	0	197 (16.6)
(), % mortality

**Table 3:** Thoracic aortic aneurysm (total; 22,982) (1) Dissection (total; 11,247)

Stanford type	Acute									Chronic								Concomitant operation					
	A					B				A				B									
Replaced site	Cases	30-day mortality			Hospital mortality	Cases	30-day mortality		Hospital mortality	Cases	30-day mortality		Hospital mortality	Cases	30-day mortality		Hospital mortality	AVP	AVR	MVP	MVR	CABG	Others
		Hospital	After discharge				Hospital	After discharge			Hospital	After discharge			Hospital	After discharge							
Ascending Ao	1934	129 (6.7)	2 (0.10)		175 (9.0)	4	1 (25.0)	0	1 (25.0)	222	6 (2.7)	0	7 (3.2)	3	0	0	0	56	130	18	11	84	35
Aortic Root	188	22 (11.7)	0		25 (13.3)	0	0	0	0	94	3 (3.2)	1 (1.1)	5 (5.3)	5	0	0	0	29	194	2	1	66	6
Arch	2092	143 (6.8)	1 (0.05)		190 (9.1)	21	0	0	0	393	5 (1.3)	0	11 (2.8)	176	9 (5.1)	0	13 (7.4)	66	133	11	11	124	32
Aortic root + asc. Ao. + Arch	170	20 (11.8)	0		27 (15.9)	1	0	0	0	60	2 (3.3)	0	3 (5.0)	7	1 (14.3)	0	1 (14.3)	31	151	2	0	56	0
Descending Ao	20	0	0		1 (5.0)	33	4 (12.1)	0	5 (15.2)	73	1 (1.4)	0	3 (4.1)	201	3 (1.5)	0	9 (4.5)	2	2	0	0	2	0
Thoracoabdominal	2	0	0		0	19	2 (10.5)	0	4 (21.1)	55	4 (7.3)	0	6 (10.9)	163	12 (7.4)	0	18 (11.0)	0	0	0	0	0	0
Simple TEVAR	105	9 (8.6)	0		12 (11.4)	450	37 (8.2)	1 (0.2)	43 (9.6)	251	2 (0.8)	1 (0.4)	4 (1.6)	1186	14 (1.2)	1 (0.1)	22 (1.9)	0	1	0	0	0	1
Open SG with BR	1350	112 (8.3)	2 (0.15)		135 (10.0)	59	3 (5.1)	0	6 (10.2)	193	4 (2.1)	1 (0.5)	8 (4.1)	229	8 (3.5)	0	11 (4.8)	48	132	7	3	103	13
Open SG without BR	526	50 (9.5)	0		62 (11.8)	21	2 (9.5)	0	2 (9.5)	74	1 (1.4)	0	3 (4.1)	85	3 (3.5)	0	4 (4.7)	17	47	3	1	34	2
Arch TEVAR with BR	20	0	0		0	146	11 (7.5)	0	13 (8.9)	67	1 (1.5)	0	2 (3.0)	431	5 (1.2)	0	7 (1.6)	0	0	0	0	0	0
Thoracoabdominal TEVAR with BR	3	0	0		0	3	0	0	0	8	0	0	0	23	2 (8.7)	0	3 (13.0)	0	0	0	0	0	0
Other	6	2 (33.3)	0		2 (33.3)	21	4 (19.0)	0	6 (28.6)	19	1 (5.3)	0	1 (5.3)	35	0	0	1 (2.9)	0	0	0	0	0	0
Total	6416	323 (5.0)	5 (0.08)		629 (9.8)	778	64 (8.2)	1 (0.1)	80 (10.3)	1509	30 (2.0)	3 (0.2)	53 (3.5)	2544	57 (2.2)	1 (0.0)	89 (3.5)	249	790	43	27	469	89
(), % mortality																							
*Ao* aorta; *AVP* aortic valve repair; *AVR* aortic valve replacement; *MVP* mitral valve repair; *MVR* mitral valve replacement;* CABG* coronary artery bypass grafting; *TEVAR* thoracic endovascular aortic (aneurysm) repair																							
Acute, within 2 weeks from the onset																							

(2) Non-dissection (total; 11,735)														
Replaced site	Unruptured				Ruptured				Concomitant operation					
	Cases	30-day mortality		Hospital mortality	Cases	30-day mortality		Hospital mortality	AVP	AVR	MVP	MVR	CABG	Others
		Hospital	After discharge			Hospital	After discharge							
Ascending Ao	1334	20 (1.5)	3 (0.22)	38 (2.8)	53	3 (5.7)	0	9 (17.0)	37	929	69	40	154	88
Aortic Root	1114	27 (2.4)	0	42 (3.8)	50	10 (20.0)	0	14 (28.0)	285	790	67	34	148	63
Arch	2041	35 (1.7)	1 (0.05)	71 (3.5)	98	10 (10.2)	0	15 (15.3)	34	571	47	17	260	83
Aortic root + asc. Ao. + Arch	259	9 (3.5)	0	15 (5.8)	16	5 (31.3)	0	5 (31.3)	49	190	9	4	39	11
Descending Ao	276	10 (3.6)	0	22 (8.0)	36	4 (11.1)	0	6 (16.7)	1	3	1	0	10	3
Thoracoabdominal	322	12 (3.7)	0	21 (6.5)	35	7 (20.0)	0	8 (22.9)	0	0	0	0	0	0
Simple TEVAR	2417	36 (1.5)	5 (0.21)	65 (2.7)	349	49 (14.0)	2 (0.57)	64 (18.3)	0	0	0	0	1	6
Open SG with BR	1200	36 (3.0)	0	70 (5.8)	81	11 (13.6)	0	17 (21.0)	21	134	13	6	185	24
Open SG without BR	464	7 (1.5)	0	21 (4.5)	38	4 (10.5)	0	7 (18.4)	6	79	6	4	49	19
Arch TEVAR with BR	1190	19 (1.6)	3	44 (3.7)	75	9 (12.0)	0	13 (17.3)	0	3	0	0	5	4
Thoracoabdominal TEVAR with BR	98	4 (4.1)	1 (1.02)	10 (10.2)	14	1 (7.1)	0	2 (14.3)	0	0	0	0	0	0
Other	153	6 (3.9)	1 (0.65)	9 (5.9)	22	4 (18.2)	0	5 (22.7)	0	13	0	1	5	5
Total	10,868	221 (2.0)	14 (0.13)	428 (3.9)	867	117 (13.5)	2 (0.23)	165 (19.0)	433	2712	212	106	856	306
(), % mortality														
*Ao* aorta; *AVP* aortic valve repair; *AVR* aortic valve replacement; *MVP* mitral valve repair; *MVR* mitral valve replacement; *CABG* coronary artery bypass grafting; *TEVAR* thoracic endovascular aortic (aneurysm) repair

**Table 4:** Pulmonary thromboembolism (total; 185)

	Cases	30-day mortality		Hospital mortality
		Hospital	After discharge	
Acute	125	12 (9.6)	1 (0.8)	15 (12.0)
Chronic	60		0	0
Total	185	12 (6.5)	1 (0.5)	15 (8.1)

(), % mortality

**Table 5:** Implantation of VAD (total; 144)

	Cases	30-day mortality		Hospital mortality
		Hospital	After discharge	
Implantation of VAD	144	1 (0.7)	0	9 (6.3)

(), % mortality

*VAD* ventricular assist devise

**Table 6:** Heart transplantation (total; 59)

	Cases	Hospital mortality
Heart transplantation	59	0
Heart and lung transplantation	0	0
Total	59	0 (0.0)

(), % mortality

Among the 8349 procedures for congenital heart disease conducted in 2021, 6510 were open-heart surgeries, with an overall hospital mortality rate of 1.7% (Table 1). The number of surgeries for neonates and infants in 2021 significantly decreased compared to that in 2011 (3958 vs 5048); on the other hands, hospital mortality did not significantly differ compared to those in 2011 (7.1% vs 6.6% for neonates and 2.4–2.7% for infants) despite the increasing ratio of surgeries for severe cases. In 2021, atrial septal defect (1302 cases) and ventricular septal defect (1338 cases) were the most common diseases as previously reported, with patients aged ≥ 18 years accounting for 59% of atrial septal defect and ventricular septal defect surgeries.

Hospital mortality of open heart surgeriews for complex congenital heart disease within the past 10 years was as follows (2011 [5], 2016 [6], and 2021): complete atrioventricular septal defect (2.6%, 2.4%, and 2.0%); tetralogy of Fallot (0.7%, 1.6%, and 0.5%); transposition of the great arteries with the intact septum (2.5%, 4.4%, and 5.0%), ventricular septal defect (3.6%, 8.3%, and 1.7%), single ventricle (4.4%, 5.1%, and 3.6%); and hypoplastic left heart syndrome (14.3%, 7.5%, and 8.0%). Currently, right heart bypass surgery has been commonly performed (291 bidirectional Glenn procedures, excluding 23 Damus–Kaye–Stansel procedures, and 385 Fontan type procedures, including total cavopulmonary connection) with acceptable hospital mortality rates (2.4% and 0.8%). The Norwood type I procedure was performed in 104 cases, with a relatively low hospital mortality rate (11.5%) (Table 1).

Valvular heart disease procedures, excluding transcatheter procedures, were performed less than that in the previous year. Isolated aortic valve replacement/repair with/without coronary artery bypass grafting (CABG) (*n* = 8206) was 4.5% fewer than that in the previous year (*n* = 8592) and 13.4% fewer than that 5 years ago (*n* = 9472 in 2016), as opposed to the rapid increase of transcatheter aortic valve replacement (*n* = 9774 and 12,202 in 2020 and 2021). Isolated mitral valve replacement/repairs with/without CABG (*n* = 4415) was not differ compared that in the previous year (*n* = 4471) and 3.5% fewer than that 5 years ago (*n* = 4576 in 2016). Aortic and mitral valve replacement with bioprosthesis were performed in 8866 and 2183 cases, respectively. The rate at which bioprosthesis was used had dramatically increased from 30% in the early 2000s [7, 8] to 88.2% and 71.5% in 2021 for aortic and mitral positions, respectively. Additionally, CABG was performed concurrently in 18.0% of all valvular procedures (17.5% in 2011 [5] and 18.4% in 2016 [6]). Valve repair was common in mitral and tricuspid valve positions (5662 and 3875 cases, respectively) but less common in aortic valve positions (152 patients, only 1.5% of all aortic valve procedures). Mitral valve repair accounted for 64.4% of all mitral valve procedures. Hospital mortality rates for isolated valve replacement for aortic and mitral positions were 2.5% and 7.9%, respectively, but only 1.2% for mitral valve repair. Moreover, hospital mortality rates for redo isolated valve surgery for the aortic and mitral positions were 5.3% and 6.5%, respectively. Finally, overall hospital mortality rates did not significantly improve over the past 10 years (3.4% in 2011 [5], 3.4% in 2016 [6], and 3.0% in 2021) (Table 2).

Isolated CABG had been performed in 10,184 cases, accounting for only 71.4% of the procedures performed 10 years ago (*n* = 14,256 in 2011) [5]. Of the aforementioned cases, 5807 (57.0%) underwent off-pump CABG, with a success rate of 97.3%. The percentage of planned off-pump CABG in 2021 was similar to that in 2020. Hospital mortality associated with primary elective CABG procedures among 8738 cases accounted for 1.8%, which is slightly higher than that in 2011 (1.1%) [5]. Hospital mortality for primary emergency CABG among 1352 cases remained high (9.2%). The percentage of conversion from off-pump to on-pump CABG or on-pump beating-heart CABG was 2.6% among the primary elective CABG cases, with a hospital mortality rate of 5.6%. Patients with end-stage renal failure on dialysis had higher hospital mortality rates than overall mortality, regardless of surgical procedure (on-pump arrest, on-pump beating, and off-pump). This study excluded concomitant CABGs alongside other major procedures under the ischemic heart disease category but rather under other categories, such as valvular heart disease and thoracic aortic aneurysm. Accordingly, the overall number of CABGs in 2020, including concomitant CABG with other major procedures, was 15,158 (Table 2).

Arrhythmia management was primarily performed as concomitant procedures in 6720 cases, with a hospital mortality rate of 3.4%. Pacemaker and implantable cardioverter-defibrillator implantation were not included in this category (Table 2).

In 2021, 22,982 procedures for thoracic and thoracoabdominal aortae diseases were performed, among which aortic dissection and non-dissection accounted for 11,247 and 11,735, respectively. The number of surgeries for aortic dissection this year was 3.6% higher than that in the preceding year (*n* = 10,855 in 2020). Hospital mortality rates for the 6416 Stanford type A acute aortic dissections remained high (9.8%). The number of procedures for non-dissected aneurysms increased by 0.4%, with a hospital mortality rate of 5.1% for all aneurysms and 3.9% and 19.0% for unruptured and ruptured aneurysms, respectively. Thoracic endovascular aortic repair (TEVAR) has been performed for aortic diseases at an increasing rate. Stent graft placement was performed in 5230 patients with aortic dissection, including 2693 TEVARs and 2537 open stent graftings. Moreover, 1640 and 314 cases underwent TEVAR and open stent grafting for type B chronic aortic dissection, accounting for 60.9% and 12.4% of the total number of cases, respectively. Hospital mortality rates associated with simple TEVAR for type B aortic dissection were 9.6% and 1.9% for acute and chronic cases, respectively. Stent graft placement was performed in 5926 patients with non-dissected aortic aneurysms, among which 4143 were TEVARs (an 1.3% increase compared to that in 2020, *n* = 4090) and 1783 were open stent graftings (a 10.6% increase compared to that in 2020, *n* = 1612). Hospital mortality rates were 3.2% and 18.0% for TEVARs and 5.5% and 20.2% for open stenting in unruptured and ruptured aneurysms, respectively (Table 3).

### (B) General thoracic surgery

**Fig. 2:**
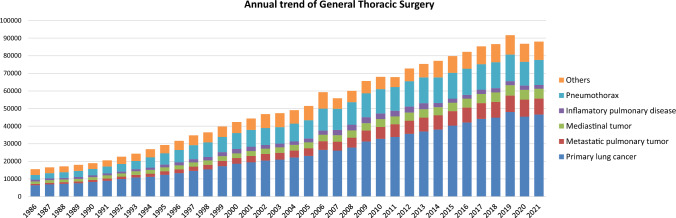
Annual trend of general thoracic surgery

The 2021 survey of general thoracic surgeries comprised 699 surgical units, with bulk data submitted via a web-based collection system established by the NCD [3]. General thoracic surgery departments reported 88,027 procedures in 2021 (Table 7), which is 2.1 times more than that in 2000 and 5834 more procedures than that in 2016 [6] (Fig. 2). It increased compared to that in 2020 (the first year of COVID-19 pandemic: 86,813) [3] by 1.4%. However it still decreased by 3.9% compared to that of 2019 (before COVID-19 pandemic: 91,626) [2], mostly because of the protraction of COVID-19 pandemic, despite the steadily increase up to 2019.

**Table 7:** Total cases of general thoracic surgery during 2021

	Cases	%
Benign pulmonary tumor	2418	2.7
Primary lung cancer	46,624	53.0
Other primary malignant pulmonary tumor	405	0.5
Metastatic pulmonary tumor	9047	10.3
Tracheal tumor	90	0.1
Pleural tumor including mesothelioma	524	0.6
Chest wall tumor	716	0.8
Mediastinal tumor	5590	6.4
Thymectomy for MG without thymoma	139	0.2
Inflammatory pulmonary disease	2117	2.4
Empyema	3123	3.5
Bullous disease excluding pneumothorax	273	0.3
Pneumothorax	14,266	16.2
Chest wall deformity	282	0.3
Diaphragmatic hernia including traumatic	37	0.0
Chest trauma excluding diaphragmatic hernia	461	0.5
Lung transplantation	93	0.1
Others	1822	2.1
Total	88,027	100.0

In 2021, 46,624 procedures for primary lung cancer had been performed which increased by 2.6% compared to that of 2020 (45,436) [3], but still decreased by 3.0% compared to that of 2019 (48,052) [2], similarly to the total number of surgeries in general thoracic surgery. The number of procedures in 2021 was 2.5 times higher than that in 2000, with lung cancer procedures accounting for 53% of all general thoracic surgeries.

Information about the number of video-assisted thoracoscopic surgery (VATS), which is defined as surgical procedures using a skin incision less than 8 cm including a mini-thoracotomy (hybrid) approach, have been available since the 2015 annual report. Tables 8, 9, 11, 14, 15, 16, 18, 19, 20, 21, 22, 24, 25, and 26 present the number of VATS procedures for benign pulmonary tumors, primary lung cancer, metastatic pulmonary tumor, chest wall tumor, mediastinal tumor, thymectomy for myasthenia gravis, inflammatory pulmonary disease, empyema, descending necrotizing mediastinitis, bullous diseases, pneumothorax, diaphragmatic hernia, chest trauma and other respiratory surgeries in 2021, respectively.

**Table 8:** Benign pulmonary tumor

	Cases	30-day mortality		Hospital mortality	By VATS
		Hospital	After discharge		
1. Benign pulmonary tumor					
Hamartoma	457	0	0	1 (0.2)	435
Sclerosing hemangioma	107	0	0	0	99
Papilloma	21	0	0	0	18
Mucous gland adenoma bronchial	19	0	0	0	19
Fibroma	118	0	0	0	112
Lipoma	9	0	0	0	9
Neurogenic tumor	16	0	0	0	13
Clear cell tumor	1	0	0	0	1
Leiomyoma	16	0	0	0	15
Chondroma	4	0	0	0	4
Inflammatory myofibroblastic tumor	1	0	0	0	1
Pseudolymphoma	18	0	0	0	15
Histiocytosis	17	0	0	0	17
Teratoma	7	0	0	0	4
Others	1607	0	0	3 (0.2)	1491
Total	2418	0	0	4 (0.17)	2253

(), Mortality %

**Table 9:** Primary malignant pulmonary tumor

	Cases	30-Day mortality		Hospital mortality	VATS	Robotic surgery
		Hospital	After discharge			
2. Primary malignant pulmonary tumor	47,029	119 (0.3)	52 (0.1)	218 (0.5)	34,458	4253
Lung cancer	46,624	119 (0.3)	52 (0.1)	216 (0.5)	34,458	4253
Histological classification						
Adenocarcinoma	32,784	46 (0.1)	28 (0.09)	75 (0.2)		
Squamous cell carcinoma	8048	50 (0.6)	17 (0.2)	97 (1.2)		
Large cell carcinoma	323	0	2 (0.6)	3 (0.9)		
LCNEC	549	2 (0.4)	2 (0.4)	3 (0.5)		
Small cell carcinoma	901	3 (0.3)	2 (0.2)	6 (0.7)		
Adenosquamous carcinoma	541	2 (0.4)	0	5 (0.9)		
Carcinoma with pleomorphic, sarcomatoid or sarcomatous elements	520	8 (1.5)	0	14 (2.7)		
Carcinoid	226	0	0	0		
Carcinomas of salivary-gland type	46	0	0	0		
Unclassified	36	0	0	0		
Multiple lung cancer	2257	5 (0.2)	1 (0.0)	9 (0.4)		
Others	358	3 (0.8)	0	4 (1.1)		
Operative procedure						
Wedge resection	8683	14 (0.2)	8 (0.1)	22 (0.3)	7982	18
Segmental excision	6781	8 (0.1)	4 (0.06)	17 (0.3)	5438	619
(Sleeve segmental excision)	14	0	0	0	11	0
Lobectomy	30,682	89 (0.3)	39 (0.13)	160 (0.5)	20,852	3609
(Sleeve lobectomy)	351	2 (0.6)	1 (0.3)	8 (2.3)	46	8
Pneumonectomy	205	5 (2.4)	0	12 (5.9)	22	2
(Sleeve pneumonectomy)	6	0	0	0	0	0
Other bronchoplasty	30	1 (3.3)	0	2 (6.7)	1	0
Pleuropneumonectomy	2	0	0	0	1	0
Others	206	2 (1.0)	1 (0.5)	3 (1.5)	133	4
Multiple incision for multiple lung cancer	35	0	0	1 (2.9)	29	1
Sarcoma	54	0	0	1 (1.9)		
AAH	103	0	0	0		
Lymphoma	197	0	0	1 (0.5)		
Others	51	0	0	0		

(), Mortality %

A total of 2418 procedures for benign pulmonary tumors had been conducted in 2021 (Table 8). Hamartomas were the most frequent benign pulmonary tumors diagnosed, with 2253 patients (93%) undergoing VATS.

Tables 9 and 10 show additional information on primary malignant pulmonary tumors. Accordingly, the most frequently diagnosed lung cancer subtype was adenocarcinoma (71% of all lung cancers), followed by squamous cell carcinoma (17%). Sublobar resection was performed in 15,464 lung cancer cases (33% of all cases) and lobectomy in 30,682 cases (66% of all cases). Sleeve lobectomy was performed in 351 cases (0.8% of all cases), while pneumonectomy was required in 205 cases (0.4% of all cases). VATS lobectomy was performed in 20,852 cases of lung cancer (68% of all lobectomy cases). RATS lobectomy was performed in 3609 cases of lung cancer (12% of all lobectomy cases). Patients aged ≥ 80 years who underwent lung cancer surgery accounted for 6912 (15%). Among those who died within 30 days postoperatively, 119 and 52 died before and after hospital discharge, respectively. Overall, 171 patients died within 30 days postoperatively (30-day mortality rate, 0.4%), while 119 died before discharge (hospital mortality rate, 0.3%). Moreover, 30-day mortality rates according to the procedure were 0.1%, 0.4%, and 2.4% for segmentectomy, lobectomy, and pneumonectomy, respectively. Interstitial pneumonia had been the leading cause of death after lung cancer surgery, followed by pneumonia, cardiovascular events and respiratory failure.

**Table 10:** Details of lung cancer operations

TNM	
c-Stage	Cases
0	2126
IA1	8867
IA2	13,972
IA3	7991
IB	4994
IIA	1582
IIB	3570
IIIA	2422
IIIB	451
IIIC	18
IVA	400
IVB	95
NA	102
Total	46,590

Sex	Cases
Male	28,363
Female	18,226
NA	0
Total	46,589

Cause of death	Cases
Cardiovascular	37
Pneumonia	73
Pyothorax	2
Bronchopleural fistula	15
Respiratory failure	22
Pulmonary embolism	7
Interstitial pneumonia	109
Brain infarction or bleeding	18
Others	136
Unknown	35
Total	454

p-Stage	Cases
0(pCR)	3308
IA1	9431
IA2	10,842
IA3	5229
IB	6560
IIA	1310
IIB	4363
IIIA	3541
IIIB	732
IIIC	11
IVA	886
IVB	90
NA	286
Total	46,589

Age (y)	Cases
< 20	20
20–29	65
30–39	235
40–49	1226
50–59	3828
60–69	11,020
70–79	23,283
80–89	6779
≥ 90	133
NA	0
Total	46,589

The procedures for metastatic pulmonary tumors performed in 2021 decreased 6.3% to 9047 cases compared to that in 2020 (9654) [3], which showed contrastive trend to primary lung cancer (Table 11). Among such procedures, the most frequent primary tumor was colorectal cancer (48% of all cases).

**Table 11:** Metastatic pulmonary tumor

	Cases	30-Day mortality		Hospital mortality	VATS	Robotic surgery
		Hospital	After discharge			
3. Metastatic pulmonary tumor	9047	5 (0.1)	9 (0.10)	10 (0.1)	8331	298
Colorectal	4307	2 (0.05)	2 (0.05)	3 (0.1)	4000	157
Hepatobiliary/Pancreatic	503	0	0	0	474	16
Uterine	530	0	0	0	483	21
Mammary	552	0	0	0	530	16
Ovarian	91	0	0	0	82	2
Testicular	50	0	0	0	45	0
Renal	733	0	0	0	687	22
Skeletal	89	0	0	0	72	5
Soft tissue	236	0	0	0	207	2
Otorhinolaryngological	469	0	2 (0.4)	1 (0.2)	434	16
Pulmonary	443	1 (0.2)	1 (0.2)	2 (0.5)	362	4
Others	1044	2 (0.2)	4 (0.4)	4 (0.4)	955	37

(), Mortality %

A total of 90 procedures for tracheal tumors, including 37, 25, and 28 cases of primary malignant, metastatic, and benign tracheal tumors, respectively, were performed in 2021. Further, 16 patients underwent sleeve resection and reconstruction (Table 12).

**Table 12:** Tracheal tumor

	Cases	30-Day mortality		Hospital mortality
		Hospital	After discharge	
4. Tracheal tumor	90	6 (6.7)	1 (1.1)	8 (8.9)
A. Primary malignant tumor				
Histological classification				
Squamous cell carcinoma	6	0	0	0
Adenoid cystic carcinoma	22	0	0	0
Mucoepidermoid carcinoma	1	0	0	0
Others	8	0	0	0
Total	37	0	0	0
B. Metastatic/invasive malignant tumor e.g. invasion of thyroid cancer				
	25	4 (16.0)	1 (4.0)	6 (24.0)
C. Benign tracheal tumor				
Papilloma	5	0	0	0
Adenoma	0	0	0	0
Neurofibroma	1	0	0	0
Chondroma	0	0	0	0
Leiomyoma	4	0	0	0
Others	18	2(11.1)	0	2(11.1)
Histology unknown	0	0	0	0
Total	28	2(7.1)	0	2(7.1)
Operative procedure				
Sleeve resection with reconstruction	16	0	0	0
Wedge with simple closure	2	0	0	0
Wedge with patch closure	0	0	0	0
Total laryngectomy with tracheostomy	0	0	0	0
Others	0	0	0	0
Unknown	0	0	0	0
Total	18	0	0	0

(), Mortality %

Overall, 524 pleural tumors had been diagnosed in 2021 (Table 13), with diffuse malignant pleural mesothelioma as the most frequent histologic diagnosis. Total pleurectomy was performed in 123 cases and extrapleural pneumonectomy in 26 cases. The 30-day mortality rate was 0% and 4% after total pleurectomy and extrapleural pneumonectomy, respectively.

**Table 13:** Tumor of pleural origin

Histological classification	Cases	30-Day mortality		Hospital mortality
		Hospital	After discharge	
Solitary fibrous tumor	101	0	0	0
Diffuse malignant pleural mesothelioma	203	3 (1.5)	0	4 (2.0)
Localized malignant pleural mesothelioma	26	0	0	0
Others	194	5 (2.6)	0	6 (3.1)
Total	524	8 (1.5)	0	10 (1.9)

Operative procedure	Cases	30-Day mortality		Hospital mortality
		Hospital	After discharge	
Extrapleural pneumonectomy	26	1 (3.8)	0	1 (3.8)
Total pleurectomy	123	0	0	0
Others	54	2 (3.7)	0	3 (5.6)
Total	203	3 (1.5)	0	4 (2.0)

(), Mortality %

Overall, 716 chest wall tumor resections had been performed in 2021, including 137, 188, and 391 cases of primary malignant, metastatic, and benign tumors, respectively (Table 14).

**Table 14:** Chest wall tumor

	Cases	30-Day mortality		Hospital mortality	VATS
		Hospital	After discharge		
6. Chest wall tumor					
Primary malignant tumor	137	0	0	0	37
Metastatic malignant tumor	188	0	2(1.1)	2(1.1)	61
Benign tumor	391	1(0.3)	0	1(0.3)	297
Total	716	1(0.1)	2(0.3)	3(0.4)	395

(), Mortality %

In 2021, 5590 mediastinal tumors were resected, which was similar to that in 2020 (5573) (Table 15) [3]. Thymic epithelial tumors, including 2174 thymomas, 380 thymic carcinomas, and 49 thymic carcinoids, were the most frequently diagnosed mediastinal tumor subtype in 2021.

**Table 15:** Mediastinal tumor

	Cases	30-Day mortality		Hospital mortality	By VATS	Robotic surgery
		Hospital	After discharge			
7. Mediastinal tumor	5590	7 (0.13)	1 (0.02)	10 (0.2)	4373	1261
Thymoma*	2174	3 (0.1)	1 (0.0)	3 (0.1)	1557	517
Thymic cancer	380	1 (0.3)	0	1 (0.3)	228	57
Thymus carcinoid	49	0	0	0	27	13
Germ cell tumor	105	1 (1.0)	0	1 (1.0)	70	19
Benign	81	1 (1.2)	0	1 (1.2)	61	17
Malignant	24	0	0	0	9	2
Neurogenic tumor	479	0	0	0	448	102
Congenital cyst	1188	0	0	1 (0.1)	1124	319
Goiter	86	0	0	0	42	7
Lymphatic tumor	164	1 (0.6)	0	1 (0.6)	130	28
Excision of pleural recurrence of thymoma	34	0	0	0	24	2
Thymolipoma	14	0	0	0	14	1
Others	917	1 (0.1)	0	3 (0.3)	709	196

(), Mortality %

A total of 505 patients underwent thymectomy for myasthenia gravis (Table 16), among which 366 procedures were associated with thymoma in 2021.

**Table 16:** Thymectomy for myasthenia gravis

	Cases	30-Day mortality		Hospital mortality	By VATS	Robotic surgery
		Hospital	After discharge			
8. Thymectomy for myasthenia gravis	505	0	0	0	352	38
With thymoma	366	0	0	0	249	5

(), Mortality %

Overall, 22,381 patients underwent procedures for non-neoplastic disease. Accordingly, 2117 patients underwent lung resection for inflammatory lung diseases (Table 17, 18), among which 428 and 270 patients were associated with mycobacterial and fungal infections, respectively. Procedures for inflammatory pseudotumor were performed in 930 cases (44%).

**Table 17:** Operations for non-neoplastic diseases: A + B + C + D + E + F + G + H + I

	Cases	30-Day mortality		Hospital mortality
		Hospital	After discharge	
9. Operations for non-neoplastic diseases	22,381	252 (1.1)	46 (0.2)	479 (2.1)

**Table 18:** A. Inflammatory pulmonary disease

	Cases	30-Day mortality		Hospital mortality	VATS
		Hospital	After discharge		
A. Inflammatory pulmonary disease	2117	8 (0.4)	3 (0.1)	14 (0.7)	1794
Tuberculous infection	29	0	0	0	21
Mycobacterial infection	428	2 (0.5)	1 (0.2)	2 (0.5)	374
Fungal infection	270	0	0	3 (1.1)	193
Bronchiectasis	41	0	0	0	29
Tuberculous nodule	58	0	0	0	50
Inflammatory pseudotumor	930	2 (0.2)	0	2 (0.2)	847
Interpulmonary lymph node	37	0	0	0	36
Others	324	4 (1.2)	2 (0.6)	7 (2.2)	244

(), Mortality %

A total of 3123 procedures were performed for empyema (Table 19), among which 2508 (80%) were acute and 615 (20%) were chronic. Further, pleural fistulas developed in 483 and 277 patients with acute and chronic empyema, respectively. The hospital mortality rate was 13% among patients with acute empyema with fistula.

**Table 19:** B. Empyema

	Cases	30-Day mortality		Hospital mortality	By VATS
		Hospital	After discharge		
Acute empyema	2508	60 (2.4)	5 (0.2)	127 (5.1)	2038
With fistula	483	32 (6.6)	2 (0.4)	64 (13.3)	235
Without fistula	2000	25 (1.3)	3 (0.2)	60 (3.0)	1780
Unknown	25	3 (12.0)	0	3 (12.0)	23
Chronic empyema	615	13 (2.1)	4 (0.7)	55 (8.9)	315
With fistula	277	5 (1.8)	2 (0.7)	33 (11.9)	81
Without fistula	299	6 (2.0)	2 (0.7)	18 (6.0)	202
Unknown	39	2 (5.1)	0	4 (10.3)	32
Total	3123	73 (2.3)	9 (0.3)	182 (5.8)	2353

(), Mortality %

Further, 94 operations were performed for descending necrotizing mediastinitis (Table 20), with a hospital mortality rate of 11%.

**Table 20:** C. Descending necrotizing mediastinitis

	Cases	30-Day mortality		Hospital mortality	VATS
		Hospital	After discharge		
C. Descending necrotizing mediastinitis	94	4 (4.3)	0	10 (10.6)	59

(), Mortality %

A total of 273 procedures were conducted for bullous diseases (Table 21), while only 14 patients underwent lung volume reduction surgery.

**Table 21:** D. Bullous diseases

	Cases	30-Day mortality		Hospital mortality	VATS
		Hospital	After discharge		
D. Bullous diseases	273	3 (1.1)	0	3 (1.1)	241
Emphysematous bulla	198	2 (1.0)	0	2 (1.0)	183
Bronchogenic cyst	7	0	0	0	6
Emphysema with LVRS	14	1 (7.1)	0	1 (7.1)	11
Others	54	0	0	0	41

(), Mortality %

*LVRS* lung volume reduction surgery

A total of 14,266 procedures were performed for pneumothorax (Table 22). Among the 10,329 procedures for spontaneous pneumothorax, 2465 (24%) were bullectomies alone, while 7217 (70%) required additional procedures, such as coverage with artificial material, as well as parietal pleurectomy. A total of 3937 procedures for secondary pneumothorax were performed, with chronic obstructive pulmonary disease (COPD) being the most prevalent associated disease (2745 cases, 70%). The hospital mortality rate for secondary pneumothorax associated with COPD was 2.4%.

**Table 22:** E. Pneumothorax

Cases	30-Day mortality		Hospital mortality	VATS
	Hospital	After discharge		
14,266	94 (0.7)	29 (0.2)	159 (1.1)	13,880

Spontaneous pneumothorax					
Operative procedure	Cases	30-Day mortality		Hospital mortality	VATS
		Hospital	After discharge		
Bullectomy	2465	4 (0.2)	1 (0.0)	7 (0.3)	2424
Bullectomy with additional procedure	7217	9 (0.1)	2 (0.03)	14 (0.2)	7123
Coverage with artificial material	7011	8 (0.1)	2 (0.03)	12 (0.2)	6924
Parietal pleurectomy	40	0	0	1 (2.5)	39
Coverage and parietal pleurectomy	63	0	0	0	61
Others	103	1 (1.0)	0	1 (1.0)	99
Others	636	7 (1.1)	1 (0.2)	8 (1.3)	584
Unknown	11	0	1 (9.1)	0	9
Total	10,329	20 (0.2)	5 (0.0)	29 (0.3)	10,140

Secondary pneumothorax					
Associated disease	Cases	30-Day mortality		Hospital mortality	VATS
		Hospital	After discharge		
COPD	2745	39 (1.4)	10 (0.4)	66 (2.4)	2625
Tumorous disease	156	11 (7.1)	4 (2.6)	16 (10.3)	147
Catamenial	200	0	0	0	199
LAM	39	0	0	0	39
Others (excluding pneumothorax by trauma)	797	24 (3.0)	8 (1.0)	48 (6.0)	730
Unknown	0	0	0	0	0

Operative procedure	Cases	30 Day mortality		Hospital mortality	VATS
		Hospital	After discharge		
Bullectomy	693	10 (1.4)	5 (0.7)	19 (2.7)	673
Bullectomy with additional procedure	2359	25 (1.1)	9 (0.4)	44 (1.9)	2285
coverage with artificial material	2265	25 (1.1)	9 (0.4)	41 (1.8)	2197
parietal pleurectomy	7	0	0	0	7
coverage and parietal pleurectomy	31	0	0	0	29
others	56	0	0	3 (5.4)	52
Others	882	39 (4.4)	8 (0.9)	67 (7.6)	776
Unknown	3	0	0	0	3
Total	3937	74 (1.9)	22 (0.6)	130 (3.3)	3737

(), Mortality %

The 2021 survey reported 282 procedures for chest wall deformity (Table 23). However, this may have been underestimated because the Nuss procedure for pectus excavatum was more likely performed in pediatric surgery centers not associated with the Japanese Association for Thoracic Surgery.

**Table 23:** F. Chest wall deformity

	Cases	30-Day mortality		Hospital mortality
		Hospital	After discharge	
F. Chest wall deformity	282	0	0	0
Funnel chest	268	0	0	0
Others	14	0	0	1 (7.1)

(), Mortality %

Surgical treatment for diaphragmatic hernia was performed in 37 patients (Table 24). This may have been underestimated because procedures may have been classified as gastrointestinal surgery.

**Table 24:** G. Diaphragmatic hernia

	Cases	30-Day mortality		Hospital mortality	VATS
		Hospital	After discharge		
G. Diaphragmatic hernia	37	0	0	0	11
Congenital	8	0	0	0	1
Traumatic	12	0	0	0	3
Others	17	0	0	0	7

(), Mortality %

The survey reported 461 procedures for chest trauma, excluding iatrogenic injuries (Table 25), with a hospital mortality rate of 7.6%.

**Table 25:** H. Chest trauma

	Cases	30-Day mortality		Hospital mortality	VATS
		Hospital	After discharge		
H. Chest trauma	461	32 (6.9)	2 (0.4)	35 (7.6)	257

(), Mortality %

Table 26 summarizes the procedures for other diseases, including 98 and 87 cases of arteriovenous malformation and pulmonary sequestration, respectively.

**Table 26:** I. Other respiratory surgery

	Cases	30-Day mortality		Hospital mortality	VATS
		Hospital	After discharge		
I. Other respiratory surgery	1728	38 (2.2)	3 (0.2)	75 (4.3)	1267
Arteriovenous malformation*	98	0	0	0	93
Pulmonary sequestration	87	0	0	0	71
Postoperative bleeding ・air leakage	553	14 (2.5)	2 (0.4)	38 (6.9)	344
Chylothorax	55	0	0	0	45
Others	935	24 (2.6)	1 (0.1)	37 (4.0)	714

(), Mortality %

A total of 93 lung transplantations were performed in 2021 (Table 27), among which 74 and 19 were from brain-dead and living-related donors, respectively. 30-day mortality for total lung transplantation was 1.1% (1/93).

**Table 27:** Lung transplantation

	Cases	30-Day mortality		Hospital mortality
		Hospital	After discharge	
Single lung transplantation from brain-dead donor	44	0	0	0
Bilateral lung transplantation from brain-dead donor	30	1 (3.3)	0	3 (10.0)
Lung transplantation from living donor	19	0	0	1 (5.3)
Total lung transplantation	93	1 (1.1)	0	4 (4.3)
Donor of living donor lung transplantation	37	0	0	0

(), Mortality %

In 2021, the number of VATS procedures increased by 1.4% from 76,073 to 77,152 compared to that of 2020 [3]with the increase of all procedures in general thoracic surgery (1.4%). The population of VATS procedures in all procedures 88% in 2021 was similar as that in 2020 (88%) (Table 28).

**Table 28:** Video-assisted thoracic surgery

	Cases	30-Day mortality		Hospital mortality
		Hospital	After discharge	
11. Video-assisted thoracic surgery	77,152	256 (0.3)	86 (0.1)	434 (0.6)

(), Mortality % (including thoracic sympathectomy 330)

A total of 590 tracheobronchoplasty procedures were performed in 2021, including 352 sleeve lobectomies, 10 carinal reconstructions and 9 sleeve pneumonectomies (Table 29). 30-day mortality for sleeve lobectomy, carinal reconstruction and sleeve lobectomy were 10, 0 and 2% respectively.

**Table 29:** Tracheobronchoplasty

	Cases	30-Day mortality		Hospital mortality
		Hospital	After discharge	
12. Tracheobronchoplasty	590	11 (1.9)	2 (0.3)	23 (3.9)
Trachea	30	0	0	0
Sleeve resection with reconstruction	19	0	0	0
Wedge with simple closure	3	0	0	0
Wedge with patch closure	0	0	0	0
Total laryngectomy with tracheostomy	0	0	0	0
Others	8	0	0	0
Carinal reconstruction	10	0	0	1 (10.0)
Sleeve pneumonectomy	9	0	0	0
Sleeve lobectomy		352	1 (0.3)	7 (2.0)
Sleeve segmental excision		15	0	0
Bronchoplasty without lung resection		16	0	1 (6.3)
Others	158	8 (5.1)	1 (0.6)	14 (8.9)

(), Mortality %

Tables 30, 31, and 32 present the details regarding pediatric surgery and combined resection of neighboring organs.

**Table 30:** Pediatric surgery

	Cases	30-Day mortality		Hospital mortality
		Hospital	After discharge	
13. Pediatric surgery	355	5 (1.4)	0	5 (1.4)

(), Mortality %

**Table 31:** Combined resection of neighboring organ(s)

	Cases	30-Day mortality		Hospital mortality
		Hospital	After discharge	
14. Combined resection of neighboring organ(s)	1229	11 (0.9)	1 (0.1)	21 (1.7)

Organ resected	Cases	30-Day mortality		Hospital mortality
		Hospital	After discharge	
A. Primary lung cancer				
Aorta	7	0	0	0
Superior vena cava	22	1 (4.5)	0	4 (18.2)
Brachiocephalic vein	7	0	0	0
Pericardium	65	0	0	2 (3.1)
Pulmonary artery	105	2 (1.9)	1 (1.0)	4 (3.8)
Left atrium	9	0	0	1 (11.1)
Diaphragm	52	0	0	0
Chest wall (including ribs)	279	5 (1.8)	0	8 (2.9)
Vertebra	9	0	0	0
Esophagus	3	0	0	0
Total	558	8 (1.4)	1 (0.2)	19 (3.4)
B. Mediastinal tumor				
Aorta	4	0	0	0
Superior vena cava	58	1 (1.7)	0	1 (1.7)
Brachiocephalic vein	111	0	0	0
Pericardium	357	2 (0.6)	0	2 (0.6)
Pulmonary artery	5	0	0	0
Left atrium	1	0	0	0
Diaphragm	40	0	0	0
Chest wall (including ribs)	17	0	0	0
Vertebra	4	0	0	0
Esophagus	4	0	0	0
Lung	457	1 (0.2)	0	1 (0.2)
Total	1058	4 (0.4)	0	4 (0.4)

(), Mortality %

**Table 32:** Operation of lung cancer invading the chest wall of the apex

	Cases	30-Day mortality		Hospital mortality
		Hospital	After discharge	
15. Operation of lung cancer invading the chest wall of the apex	588	6 (1.0)	1 (0.2)	11 (1.9)

(), Mortality %. Includes tumors invading the anterior apical chest wall and posterior apical chest wall (superior sulcus tumor, so called Pancoast type)

### (C) Esophageal surgery

In 2018, the data collection method for esophageal surgery had been modified from self-reports using questionnaire sheets following each institution belonging to the Japanese Association for Thoracic Surgery to an automatic package downloaded from the NCD in Japan. Consequently, the registry excluded data for non-surgical cases with esophageal diseases. Furthermore, data regarding the histological classification of malignant tumors, multiple primary cancers, and mortality rates for cases with combined resection of other organs could not be registered because they were not included in the NCD. Instead, detailed data regarding postoperative surgical and non-surgical complications were collected from the NCD. Moreover, data regarding surgeries for corrosive esophageal strictures and salvage surgeries for esophageal cancer had been exceptionally registered by participating institutions (Table 33).

**Table 33:** Diagnostic procedures

	Cases	30-Day mortality		Hospital mortality
		Hospital	After discharge	
Mediastinoscopic biopsy	258	0	1 (0.4)	1 (0.4)
Lung biopsy for diffuse parenchymal lung disease	634	3 (0.5)	2 (0.3)	5 (0.8)
Biopsy for lymph node, tumor and pleura	2926	27 (0.9)	20 (0.7)	48 (1.6)
Others	1494	68 (4.6)	12 (0.8)	114 (7.6)

(), Mortality %

**Fig. 3:**
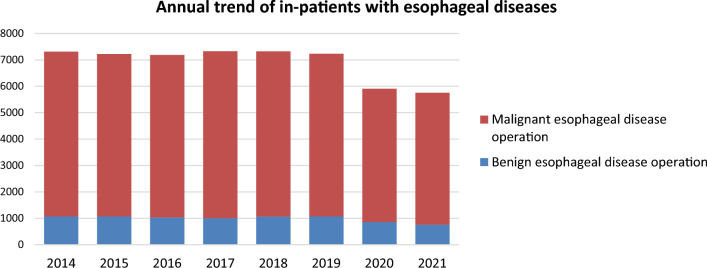
Annual trend of in-patients with esophageal diseases

Throughout 2021, 5755 patients underwent surgery for esophageal diseases (752 and 4993 for benign and malignant esophageal diseases, respectively) from institutions across Japan. Compared to 2019, there was a total decrease of 1480 cases (20.5%) observed, and a decrease of 154 cases (2.6%) compared to 2020 with a decrease of 98 cases (11.4%) in benign diseases and a decrease of 56 cases (1.1%) in malignant diseases. These significant declines which were largely influenced by the COVID-19 pandemic that began in 2020, continued even in 2021, with factors such as surgical restrictions, reduced medical visits, and postponed screenings being considered as contributing factors (Fig. 3).

Concerning benign esophageal diseases (Table 34), thoracoscopic and/or laparoscopic surgeries were performed in 89.3% (42/47), 85.8% (363/423), 97.8% (44/45), and 43.5% (54/124) of patients with esophagitis (including esophageal ulcer), hiatal hernia, benign tumors, and achalasia, respectively. Conversely, 100% (93/93) of patients with spontaneous rupture of the esophagus underwent open surgery. Hospital mortality rates within 30 postoperative days were 0.9% (4/423), 1.1% (1/93) for hiatal hernia and spontaneous rupture of the esophagus, respectively.

**Table 34:** Benign esophageal diseases

		Operation ( +)				T/L*3		
	Cases	Hospital mortality			Cases	Hospital mortality		
		~ 30 days	31–90 days	Total (including after 91days mortality)		~ 30days	31–90days	Total (including after 91days mortality)
1. Achalasia	124	0	0	0	54	0	0	0
2. Benign tumor	45	0	0	0	44	0	0	0
3. Diverticulum	25	0	0	0	5	0	0	0
4. Hiatal hernia	423	4 (0.9)	1 (0.2)	5 (1.2)	363	2 (0.6)	1 (0.3)	3 (0.8)
5. Spontaneous rupture of the esophagus	93	1 (1.1)	0	1 (1.1)	0	0	0	0
6. Esophago-tracheal fistula	5	0	0	0	0	0	0	0
7. Esophagitis, esophageal ulcer	47	0	0	0	42	0	0	0
Total	762	5 (0.7)	1 (0.1)	6 (0.8)	508	2 (0.4)	1 (0.2)	3 (0.6)

(), Mortality %

*T/L* thoracoscopic and/or laparoscopic

The most common tumor location for malignant esophageal diseases was the thoracic esophagus (Table 35). Among the cases with esophageal malignancies, esophagectomy for superficial and advanced cancers was performed in 1847 (40.0%) and 3146 (60.0%), respectively. Hospital mortality rates within 30 days after esophagectomy were 0.5% and 0.8% for patients with superficial and advanced cancer, respectively.

**Table 35:** Malignant Esophageal disease

	Operation ( +)					Thoracoscopic and/or laparscopic procedure			
	Cases	Hospital mortality			Cases	Conversion to thoracotomy	Hospital mortality		
		~ 30days	31–90days	Total (including after 91days mortality)			~ 30days	31–90days	Total (including after 91days mortality)
Location									
(1) Cervical esophagus	118	2 (1.7)	0	2 (1.7)	51	1 (2.0)	1 (2.0)	0	1 (2.0)
(2) Thoracic esophagus	4181	27 (0.6)	18 (0.4)	45 (1.1)	3788	19 (0.5)	23 (0.6)	15 (0.4)	38 (1.0)
(3) Abdominal esophagus	436	4 (0.9)	2 (0.5)	6 (1.4)	361	2 (0.6)	3 (0.8)	2 (0.6)	5 (1.4)
Total	4735	33 (0.7)	20 (0.4)	53 (1.1)	4200	22 (0.5)	27 (0.6)	17 (0.4)	44 (1.0)
Tumor depth									
(A) Superficial cancer(T1)									
(1) Transhiatal esophagectomy	4	0	0	0	0	0	0	0	0
(2) Mediastinoscopic esophagectomy and reconstruction	103	0	1 (1.0)	1 (1.0)	103	0	0	1	1 (1.0)
(3) Transthoracic (rt.) esophagectomy and reconstruction	1124	6 (0.5)	5 (0.4)	11 (1.0)	1043	4 (0.4)	6 (0.6)	5 (0.5)	11 (1.1)
(4) Transthoracic (lt.) esophagectomy and reconstruction	31	0	0	0	26	0	0	0	0
(5) Cervical esophageal resection and reconstruction	25	2 (8.0)	0	2 (8.0)	0	0	0	0	0
(6) Robot-assisted esophagectomy and reconstruction	424	1 (0.2)	0	1 (0.2)	423	0	1 (0.2)	0	1 (0.2)
(7) Others	17	0	0	0	0	0	0	0	0
(8) Esophagectomy without reconstruction	119	0	0	0	0	0	0	0	0
subtotal	1847	9 (0.5)	6 (0.3)	15 (0.8)	1595	4 (0.3)	7 (0.4)	6 (0.4)	13 (0.8)
(B)Advanced cancer(T2-T4)									
(1) Transhiatal esophagectomy	7	0	0	0	0	0	0	0	0
(2) Mediastinoscopic esophagectomy and reconstruction	129	0	1 (0.8)	1 (0.8)	129	0	0	1 (0.8)	1 (0.8)
(3) Transthoracic (rt.) esophagectomy and reconstruction	2099	17 (0.8)	10 (0.5)	27 (1.3)	1791	18 (1.0)	14 (0.8)	7 (0.4)	21 (1.2)
(4) Transthoracic (lt.) esophagectomy and reconstruction	58	1 (1.7)	0	1 (1.7)	33	0	0	0	0
(5) Cervical esophageal resection and reconstruction	52	0	0	0	0	0	0	0	0
(6) Robot-assisted esophagectomy and reconstruction	644	6 (0.9)	3 (0.5)	9 (1.4)	643	0	6 (0.9)	3 (0.5)	3 (0.5)
(7) Others	18	0	0	0	0	0	0	0	0
(8) Esophagectomy without reconstruction	139	0	0	0	0	0	0	0	0
Subtotal	3146	24 (0.8)	14 (0.4)	38 (1.2)	2596	18 (0.7)	20 (0.8)	11 (0.4)	25 (1.0)
Total	4993	33 (0.7)	20 (0.4)	53 (1.1)	4191	22 (0.5)	27 (0.6)	17 (0.4)	38 (0.9)

	Cases	Overall morbidity	Morbidity ≥ CD III	Surgical complications					
				Surgical site infection			Anastomotic leakage	Recurrent nerve palsy	Wound dehiscence
				Superficial incision	Deep incision	Organ space			
Location									
(1) Cervical esophagus	118	61 (51.7)	29 (24.6)	10 (8.5)	5 (4.2)	11 (9.3)	15 (12.7)	17 (14.4)	3 (2.5)
(2) Thoracic esophagus	4181	2340 (56.0)	968 (23.2)	272 (6.5)	143 (3.4)	316 (7.6)	532 (12.7)	594 (14.2)	45 (1.1)
(3) Abdominal esophagus	436	215 (49.3)	96 (22.0)	24 (5.5)	13 (3.0)	41 (9.4)	57 (13.1)	45 (10.3)	3 (0.7)
Total	4735	2616 (55.2)	1093 (23.1)	306 (6.5)	161 (3.4)	368 (7.8)	604 (12.8)	656 (13.9)	51 (1.1)
Tumor depth									
(A)Superficial cancer(T1)									
(1) Transhiatal esophagectomy	4	1 (25.0)	0	0	0	0	0	0	0
(2) Mediastinoscopic esophagectomy and reconstruction	103	64 (62.1)	31 (30.1)	7 (6.8)	3 (2.9)	13 (12.6)	23 (22.3)	28 (27.2)	0
(3) Transthoracic (rt.) esophagectomy and reconstruction	1124	619 (55.1)	217 (19.3)	63 (5.6)	34 (3.0)	80 (7.1)	161 (14.3)	153 (13.6)	10 (0.9)
(4) Transthoracic (lt.) esophagectomy and reconstruction	31	12 (38.7)	7 (22.6)	4 (12.9)	1 (3.2)	1 (3.2)	2 (6.5)	3 (9.7)	0
(5) Cervical esophageal resection and reconstruction	25	12 (48.0)	7 (28.0)	3 (12.0)	0	1 (4.0)	1 (4.0)	4 (16.0)	0
(6) Robot-assisted esophagectomy and reconstruction	424	200 (47.2)	85 (20.0)	20 (4.7)	11 (2.6)	27 (6.4)	53 (12.5)	53 (12.5)	2 (0.5)
(7) Others	17	4 (23.5)	2 (11.8)	1 (5.9)	1 (5.9)	3 (17.6)	4 (23.5)	4 (23.5)	0
(8) Esophagectomy without reconstruction	119	0	0	0	0		0	0	0
Subtotal	1847	912 (49.4)	349 (18.9)	98 (5.3)	50 (2.7)	125 (6.8)	244 (13.2)	245 (13.3)	12 (0.6)
(B) Advanced cancer (T2–T4)									
(1) Transhiatal esophagectomy	7	4 (57.1)	1 (14.3)	1 (14.3)	0	0	0	0	0
(2) Mediastinoscopic esophagectomy and reconstruction	129	76 (58.9)	28 (21.7)	14 (10.9)	2 (1.6)	4 (3.1)	15 (11.6)	26 (20.2)	0
(3) Transthoracic (rt.) esophagectomy and reconstruction	2099	1213 (57.8)	529 (25.2)	144 (6.9)	84 (4.0)	173 (8.2)	263 (12.5)	271 (12.9)	35 (1.7)
(4) Transthoracic (lt.) esophagectomy and reconstruction	58	24 (41.4)	7 (12.1)	2 (3.4)	2 (3.4)	4 (6.9)	3 (5.2)	4 (6.9)	0
(5) Cervical esophageal resection and reconstruction	52	33 (63.5)	13 (25.0)	5 (9.6)	4 (7.7)	5 (9.6)	8 (15.4)	12 (23.1)	2 (3.8)
(6) Robot-assisted esophagectomy and reconstruction	644	345 (53.6)	160 (24.8)	41 (6.4)	19 (3.0)	53 (8.2)	66 (10.2)	102 (15.8)	2 (0.3)
(7) Others	18	9 (50.0)	6 (33.3)	1 (5.6)	0	4 (22.2)	5 (27.8)	0	0
(8) Esophagectomy without reconstruction	139	0	0	0	0	0	0	0	0
subtotal	3146	1704 (54.2)	744 (23.6)	208 (6.6)	111 (3.5)	243 (7.7)	360 (11.4)	415 (13.2)	39 (1.2)
Total	4993	2616 (52.4)	1093 (21.9)	306 (6.1)	161 (3.2)	368 (7.4)	604 (12.1)	660 (13.2)	51 (1.0)

	Cases	Nonsurgical complications									Readmission within 30d	Reoperation within 30d
		Pneumonia	Unplanned intubation	Prolonged ventilation > 48 h	Pulmonary embolism	Atelectasis	Renal failure	CNS events	Cardiac events	Septic shock		
Location												
(1) Cervical esophagus	118	14 (11.9)	7 (5.9)	13 (11.0)	1 (0.8)	3 (2.5)		1 (0.8)	2 (1.7)	0	1 (0.8)	15 (12.7)
(2) Thoracic esophagus	4181	670 (16.0)	162 (3.9)	166 (4.0)	39 (0.9)	187 (4.5)	16 (0.4)	16 (0.4)	18 (0.4)	29 (0.7)	104 (2.5)	246 (5.9)
(3) Abdominal esophagus	436	50 (11.5)	10 (2.3)	18 (4.1)	6 (1.4)	27 (6.2)	3 (0.7)	2 (0.5)	1 (0.2)	4 (0.9)	5 (1.1)	25 (5.7)
Total	4735	734 (15.5)	179 (3.8)	197 (4.2)	46 (1.0)	217 (4.6)	19 (0.4)	19 (0.4)	21 (0.4)	33 (0.7)	110 (2.3)	286 (6.0)
Tumor depth												
(A) Superficial cancer (T1)												
(1) Transhiatal esophagectomy	4	0	0	0	0	0	0	0	0	0	0	0
(2) Mediastinoscopic esophagectomy and reconstruction	103	13 (12.6)	3 (2.9)	4 (3.9)	1 (1.0)	4 (3.9)	0	0	2 (1.9)	1 (1.0)	3 (2.9)	5 (4.9)
(3) Transthoracic (rt.) esophagectomy and reconstruction	1124	163 (14.5)	42 (3.7)	37 (3.3)	5 (0.4)	51 (4.5)	2 (0.2)	7 (0.6)	4 (0.4)	8 (0.7)	23 (2.0)	68 (6.0)
(4) Transthoracic (lt.) esophagectomy and reconstruction	31	4 (12.9)	1 (3.2)	1 (3.2)	0	3 (9.7)	1 (3.2)	0	0	0	1 (3.2)	2 (6.5)
(5) Cervical esophageal resection and reconstruction	25	5 (20.0)	2 (8.0)	2 (8.0)	0	1 (4.0)	0	0	0	1 (4.0)	0	3 (12.0)
(6) Robot-assisted esophagectomy and reconstruction	424	57 (13.4)	10 (2.4)	12 (2.8)	6 (1.4)	13 (3.1)	0	1 (0.2)	1 (0.2)	1 (0.2)	11 (2.6)	31 (7.3)
(7) Others	17	0	0	0	0	0	0	0	0	0	1 (5.9)	2 (11.8)
(8) Esophagectomy without reconstruction	119	0	0	0	0	0	0	0	0	0	0	0
subtotal	1847	242 (13.1)	58 (3.1)	56 (3.0)	12 (0.6)	72 (3.9)	3 (0.2)	8 (0.4)	7 (0.4)	11 (0.6)	39 (2.1)	111 (6.0)
(B)Advanced cancer (T2–T4)												
(1) Transhiatal esophagectomy	7	2 (28.6)	0	0	0	0	0	0	0	0	0	0
(2) Mediastinoscopic esophagectomy and reconstruction	129	25 (19.4)	2 (1.6)	3 (2.3)	1 (0.8)	8 (6.2)	0	0	1 (0.8)	0	4 (3.1)	3 (2.3)
(3) Transthoracic (rt.) esophagectomy and reconstruction	2099	354 (16.9)	91 (4.3)	106 (5.1)	23 (1.1)	101 (4.8)	11 (0.5)	9 (0.4)	10 (0.5)	14 (0.7)	50 (2.4)	130 (6.2)
(4) Transthoracic (lt.) esophagectomy and reconstruction	58	8 (13.8)	3 (5.2)	3 (5.2)	0	2 (3.4)	1 (1.7)	0	0	1 (1.7)	1 (1.7)	2 (3.4)
(5) Cervical esophageal resection and reconstruction	52	5 (9.6)	4 (7.7)	5 (9.6)	1 (1.9)	0	0	0	0	0	0	5 (9.6)
(6) Robot-assisted esophagectomy and reconstruction	644	96 (14.9)	21 (3.3)	24 (3.7)	9 (1.4)	33 (5.1)	4 (0.6)	2 (0.3)	1 (0.2)	7 (1.1)	17 (2.6)	35 (5.4)
(7) Others	18	2 (11.1)	0	0	0	1 (5.6)	0	0	2 (11.1)	0	0	0
(8) Esophagectomy without reconstruction	139	0	0	0	0	0	0	0	0	0	0	0
Subtotal	3146	492 (15.6)	121 (3.8)	141 (4.5)	34 (1.1)	145 (4.6)	16 (0.5)	11(0.3)		22 (0.7)	72 (2.3)	175 (5.6)
Total	4993	734 (14.7)	179 (3.6)	197 (3.9)	46 (0.9)	217 (4.3)	19 (0.4)	19 (0.4)	21 (0.4)	33 (0.7)	111 (2.2)	286 (5.7)

**Fig. 4:**
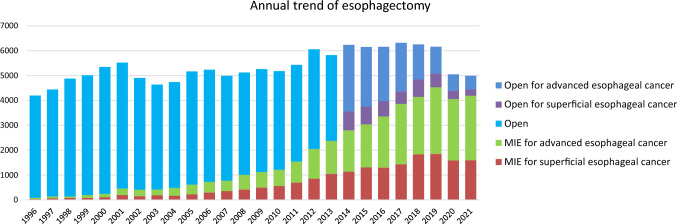
Annual trend of esophagectomy

Among esophagectomy procedures, transthoracic esophagectomy via right thoracotomy or right thoracoscopy was most commonly adopted for patients with superficial (1124/1847, 60.9%) and advanced cancer (2099/3146, 66.7%) (Table 35). Transhiatal esophagectomy, which is commonly performed in Western countries, was adopted in only 4 (0.2%) and 7 (0.2%) patients with superficial and advanced cancer who underwent esophagectomy in Japan, respectively. Minimally invasive esophagectomy (MIE) including thoracoscopic and/or laparoscopic esophagectomy, robot-assisted esophagectomy and mediastinoscopic esophagectomy was utilized in 1595 (86.3%) and 2596 (82.5%) patients with superficial and advanced cancer, respectively. Incidence of MIE for superficial or advanced cancer have been increasing, whereas that of open surgery, especially for advanced cancer, has been decreasing annually (Fig. 4). Although mediastinoscopic esophagectomy was performed only for 103 (5.6%) and 129 (4.1%) patients with superficial and advanced esophageal cancer, respectively. Robot-assisted esophagectomy has been remarkably increased since 2018 when the insurance approval was obtained in Japan, and performed for 424 (23.0%) and 624 (20.5%) patients with superficial and advanced esophageal cancer, respectively in 2021. Patients who underwent robot-assisted surgery are increasing for both superficial and advancer esophageal cancers (18.8% and 34.4% increases compared to that in 2020, respectively). Hospital mortality rates within 30 days after MIE were 0.4% and 0.8% for patients with superficial and advanced cancer, respectively (Table 35).

Detailed data collection regarding postoperative surgical and non-surgical complications was initiated in 2018. Overall, 1093 (21.9%) of 4993 patients developed grade III or higher complications based on the Clavien–Dindo classification in 2021 (Table 35). The incidence of grade III or higher complications was relatively higher in cervical esophageal cancer compared to thoracic or abdominal esophageal cancer. Among surgical complications in patients with advanced esophageal cancer, anastomotic leakage and recurrent nerve palsy occurred in 12.5% and 12.9% of the patients who underwent right transthoracic esophagectomy, in 10.2% and 15.8% of those who underwent robot-assisted esophagectomy, and in 11.6% and 20.2% of those who underwent mediastinoscopic esophagectomy, respectively. Among non-surgical postoperative complications, pneumonia occurred in 14.7% of the patients, 3.6% of whom underwent unplanned intubation. Postoperative pulmonary embolism occurred in 0.9% of the patients. These complication rates, including the others, were similar to those in 2020.

We aim to continue our efforts in collecting comprehensive survey data through more active collaboration with the Japan Esophageal Society and other related institutions, with caution due to the impact of COVID-19 pandemic.

## Data Availability

Based on the data use policy of JATS, data access is approved through assessment by the JATS: Committee for Scientific Affairs. Those interested in using the data should contact the JATS: Committee for Scientific Affairs(survey@jpats.org) to submit a proposal. The use of the data is granted for the approved study proposals.
