# Correction: Thoracic and cardiovascular surgeries in Japan during 2021: Annual report by the Japanese Association for Thoracic Surgery

**DOI:** 10.1007/s11748-024-02113-y

**Published:** 2024-12-30

**Authors:** Committee for Scientific Affairs, The Japanese Association for Thoracic Surgery, Naoki Yoshimura, Yukio Sato, Hiroya Takeuchi, Tomonobu Abe, Shunsuke Endo, Yasutaka Hirata, Michiko Ishida, Hisashi Iwata, Takashi Kamei, Nobuyoshi Kawaharada, Shunsuke Kawamoto, Kohji Kohno, Hiraku Kumamaru, Kenji Minatoya, Noboru Motomura, Rie Nakahara, Morihito Okada, Hisashi Saji, Aya Saito, Masanori Tsuchida, Kenji Suzuki, Hirofumi Takemura, Tsuyoshi Taketani, Yasushi Toh, Wataru Tatsuishi, Hiroyuki Yamamoto, Takushi Yasuda, Masayuki Watanabe, Goro Matsumiya, Yoshiki Sawa, Hideyuki Shimizu, Masayuki Chida

**Affiliations:** 1 Committee for Scientific Affairs, The Japanese Association for Thoracic Surgery, Tokyo, Japan; 2 Department of Thoracic and Cardiovascular Surgery, Graduate School of Medicine, University of Toyama, Toyama, Japan; 3 Department of Thoracic Surgery, University of Tsukuba, Tsukuba, Japan; 4 Department of Surgery, Hamamatsu University School of Medicine, Shizuoka, Japan; 5 Division of Cardiovascular Surgery, Department of General Surgical Science, Gunma University, Maebashi, Japan; 6 Thoracic Surgery, Jichi Medical University Saitama Medical Center, Omiya, Japan; 7 Department of Cardiac Surgery, The University of Tokyo Hospital, Tokyo, Japan; 8 Cardiac Surgery, Handa City Hospita, Aichi, Japan; 9 Department of General Thoracic Surgery, Gifu University Hospital, Gifu, Japan; 10 Department of Surgery, Graduate School of Medicine, Tohoku University, Sendai, Japan; 11 Department of Cardiovascular Surgery, Sapporo Medical University School of Medicine, Sapporo, Japan; 12 Department of Cardiovascular Surgery, Tohoku Medical and Pharmaceutical University Hospital, Sendai, Japan; 13 Department of Gastrointestinal Tract Surgery, Fukushima Medical University, Fukushima, Japan; 14 Department of Healthcare Quality Assessment, Graduate School of Medicine, The University of Tokyo, Tokyo, Japan; 15 Department of Cardiovascular Surgery, Graduate School of Medicine, Kyoto University, Kyoto, Japan; 16 Department of Cardiovascular Surgery, Toho University Sakura Medical Center, Chiba, Japan; 17 Division of Thoracic Surgery, Tochigi Cancer Center, Tochigi, Japan; 18 Surgical Oncology, Hiroshima University, Hiroshima, Japan; 19 Department of Chest Surgery, St. Marianna University School of Medicine, Kawasaki, Japan; 20 Department of Surgery, Graduate School of Medicine, Yokohama City University, Yokohama, Japan; 21 Division of Thoracic and Cardiovascular Surgery, Niigata University Graduate School of Medical and Dental Sciences, Niigata, Japan; 22 Department of General Thoracic Surgery, Juntendo University School of Medicine, Tokyo, Japan; 23 Department of Cardiovascular Surgery, Kanazawa University, Kanazawa, Japan; 24 Department of Cardiovascular Surgery, Mitsui Memorial Hospital, Tokyo, Japan; 25 Department of Gastroenterological Surgery, National Hospital Organization Kyushu Cancer Center, Fukuoka, Japan; 26 Department of Surgery, Faculty of Medicine, Kindai University, Osaka, Japan; 27 Department of Gastroenterological Surgery, Cancer Institute Hospita, Tokyo, Japan; 28 Department of Cardiovascular Surgery, Chiba University Graduate School of Medicine, Chiba, Japan; 29 Graduate School of Medicine, Osaka Police Hospital, Osaka University, Osaka, Japan; 30 Department of Cardiovascular Surgery, Keio University, Tokyo, Japan; 31 Department of General Thoracic Surgery, Dokkyo Medical University, Tochigi, Japan

**Correction: General Thoracic and Cardiovascular Surgery (2024) 72:254–291** 10.1007/s11748-023-01997-6

In the original publication of the article [1], the number of 30-day hospital mortality and 30-day hospital mortality rate for the Stanford type A acute aortic dissections in “Table 3 (1) Dissection” were published incorrectly. The corrected part of the table is given in this Correction. The corrected number of 30-day hospital mortality was 487, and hospital mortality rate was 7.6%, respectively.

**Table 3:** Thoracic aortic aneurysm (total; 22,982) (1) Dissection (total; 11,247)

Stanford type	Acute								Chronic								Concomitant operation					
	A				B				A				B									
Replaced site	Cases	30-day mortality		Hospital mortality	Cases	30-day mortality		Hospital mortality	Cases	30-day mortality		Hospital mortality	Cases	30-day mortality		Hospital mortality	AVP	AVR	MVP	MVR	CABG	Others
		Hospital	After discharge			Hospital	After discharge			Hospital	After discharge			Hospital	After discharge							
Ascending Ao	1934	129 (6.7)	2 (0.10)	175 (9.0)	4	1 (25.0)	0	1 (25.0)	222	6 (2.7)	0	7 (3.2)	3	0	0	0	56	130	18	11	84	35
Aortic Root	188	22 (11.7)	0	25 (13.3)	0	0	0	0	94	3 (3.2)	1 (1.1)	5 (5.3)	5	0	0	0	29	194	2	1	66	6
Arch	2092	143 (6.8)	1 (0.05)	190 (9.1)	21	0	0	0	393	5 (1.3)	0	11 (2.8)	176	9 (5.1)	0	13 (7.4)	66	133	11	11	124	32
Aortic root + asc. Ao. + Arch	170	20 (11.8)	0	27 (15.9)	1	0	0	0	60	2 (3.3)	0	3 (5.0)	7	1 (14.3)	0	1 (14.3)	31	151	2	0	56	0
Descending Ao	20	0	0	1 (5.0)	33	4 (12.1)	0	5 (15.2)	73	1 (1.4)	0	3 (4.1)	201	3 (1.5)	0	9 (4.5)	2	2	0	0	2	0
Thoracoabdominal	2	0	0	0	19	2 (10.5)	0	4 (21.1)	55	4 (7.3)	0	6 (10.9)	163	12 (7.4)	0	18 (11.0)	0	0	0	0	0	0
Simple TEVAR	105	9 (8.6)	0	12 (11.4)	450	37 (8.2)	1 (0.2)	43 (9.6)	251	2 (0.8)	1 (0.4)	4 (1.6)	1186	14 (1.2)	1 (0.1)	22 (1.9)	0	1	0	0	0	1
Open SG with BR	1350	112 (8.3)	2 (0.15)	135 (10.0)	59	3 (5.1)	0	6 (10.2)	193	4 (2.1)	1 (0.5)	8 (4.1)	229	8 (3.5)	0	11 (4.8)	48	132	7	3	103	13
Open SG without BR	526	50 (9.5)	0	62 (11.8)	21	2 (9.5)	0	2 (9.5)	74	1 (1.4)	0	3 (4.1)	85	3 (3.5)	0	4 (4.7)	17	47	3	1	34	2
Arch TEVAR with BR	20	0	0	0	146	11 (7.5)	0	13 (8.9)	67	1 (1.5)	0	2 (3.0)	431	5 (1.2)	0	7 (1.6)	0	0	0	0	0	0
Thoracoabdominal TEVAR with BR	3	0	0	0	3	0	0	0	8	0	0	0	23	2 (8.7)	0	3 (13.0)	0	0	0	0	0	0
Other	6	2 (33.3)	0	2 (33.3)	21	4 (19.0)	0	6 (28.6)	19	1 (5.3)	0	1 (5.3)	35	0	0	1 (2.9)	0	0	0	0	0	0
Total	6416	487 (7.6)	5 (0.08)	629 (9.8)	778	64 (8.2)	1 (0.1)	80 (10.3)	1509	30 (2.0)	3 (0.2)	53 (3.5)	2544	57 (2.2)	1 (0.0)	89 (3.5)	249	790	43	27	469	89

(), % mortality

*Ao* aorta, *AVP* aortic valve repair, *AVR* aortic valve replacement, *MVP* mitral valve repair, *MVR* mitral valve replacement, *CABG* coronary artery bypass grafting, *TEVAR* thoracic endovascular aortic (aneurysm) repair

Acute, within 2 weeks from the onset

## References

[1] Yoshimura N, Sato Y, Takeuchi H, et al. Thoracic and cardiovascular surgery in Japan during 2021—Annual report by the Japanese Association for Thoracic Surgery. Gen Thorac Cardiovasc Surg. 2024;72:254–91. 38421591 10.1007/s11748-023-01997-6 PMC10955033

